# Simulation for skills training in neurosurgery: a systematic review, meta-analysis, and analysis of progressive scholarly acceptance

**DOI:** 10.1007/s10143-020-01378-0

**Published:** 2020-09-18

**Authors:** Joseph Davids, Susruta Manivannan, Ara Darzi, Stamatia Giannarou, Hutan Ashrafian, Hani J Marcus

**Affiliations:** 1 Department of Neurosurgery, National Hospital for Neurology and Neurosurgery, Queen Square, Holborn, London, WC1N 3BG UK; 2 Imperial College Healthcare NHS Trust, St Mary’s Praed St, Paddington, London, W2 1NY UK; 3 Department of Neurosurgery, Southampton University NHS Trust, Tremona Road, Southampton, SO16 6YD UK

**Keywords:** Simulation, Virtual reality, Augmented reality, Neurosurgery, Meta-analysis, Progressive scholarly acceptance

## Abstract

At a time of significant global unrest and uncertainty surrounding how the delivery of clinical training will unfold over the coming years, we offer a systematic review, meta-analysis, and bibliometric analysis of global studies showing the crucial role simulation will play in training. Our aim was to determine the types of simulators in use, their effectiveness in improving clinical skills, and whether we have reached a point of global acceptance. A PRISMA-guided global systematic review of the neurosurgical simulators available, a meta-analysis of their effectiveness, and an extended analysis of their progressive scholarly acceptance on studies meeting our inclusion criteria of simulation in neurosurgical education were performed. Improvement in procedural knowledge and technical skills was evaluated. Of the identified 7405 studies, 56 studies met the inclusion criteria, collectively reporting 50 simulator types ranging from cadaveric, low-fidelity, and part-task to virtual reality (VR) simulators. In all, 32 studies were included in the meta-analysis, including 7 randomised controlled trials. A random effects, ratio of means effects measure quantified statistically significant improvement in procedural knowledge by 50.2% (ES 0.502; CI 0.355; 0.649, *p* < 0.001), technical skill including accuracy by 32.5% (ES 0.325; CI − 0.482; − 0.167, *p* < 0.001), and speed by 25% (ES − 0.25, CI − 0.399; − 0.107, *p* < 0.001). The initial number of VR studies (*n* = 91) was approximately double the number of refining studies (*n* = 45) indicating it is yet to reach progressive scholarly acceptance. There is strong evidence for a beneficial impact of adopting simulation in the improvement of procedural knowledge and technical skill. We show a growing trend towards the adoption of neurosurgical simulators, although we have not fully gained progressive scholarly acceptance for VR-based simulation technologies in neurosurgical education.

**Electronic supplementary material:**

The online version of this article (10.1007/s10143-020-01378-0) contains supplementary material, which is available to authorized users.

## Introduction

Rapid technological advancements have created opportunities to overcome global challenges in neurosurgical training delivery in the twenty-first century [19, 29–31, 39, 50, 51, 60, 62, 81–83, 97, 98, 101, 106, 110, 111]. Mounting pressures on training time due to consensus-driven working time directives in the USA and Europe have been highlighted as responsible for skills acquisition deficits [21, 22, 26, 41, 56, 57, 65, 71, 73, 82, 91, 109, 126].

Neurosurgery is a high-risk, high-stakes specialty with little margin for error. Standardising the expertise and training of neurosurgeons to ensure the highest quality of care and to minimise patient safety concerns is vital for this growing global specialty [23]. Up to 22.6 million patients suffer from neurological pathologies that warrant the expertise of a neurosurgeon with 13.8 million requiring surgery, but 5 million are unable to undergo the required surgical intervention [23]. There is therefore an argument for a much needed and skilled global neurosurgical workforce. The traditional route to managing this need for education in neurosurgery has been typically craft-based and ad hoc, but more recently, there have been some efforts to derive training through modern educational approaches of simulation [126].

High-fidelity physically immersive simulations have gained widespread adoption in neurosurgical education in recent decades [14, 18, 33, 40, 46, 85, 86]. The use of realistic models designed to closely mimic the clinical situation under scrutiny is gradually supplanting cadaveric methods [6, 37, 38, 123]. In concert, virtual, computer engineered photorealistic and 3D-printed technology for simulation have also seen an accelerated growth in adoption for subspecialty areas of neurosurgical education such as neurovascular aneurysmal surgery—also with increasing levels of fidelity [98].

A growing variety of simulators such as the ImmersiveTouch, VIST, ANGIO Mentor, ROBOSIM, SIMONT, NeuroSIM, 3D printed models as well as mobile, augmented reality (AR), virtual reality (VR), and mixed reality simulator platforms are now available for different neurosurgical subspecialties [11] [76] [84, 117–119]. Most have been previously appraised for validity and newer types continue to appear on the market [76] [84, 117–119]. Cumulative evidence also supports the development and use of virtual simulators with haptic feedback in neurosurgical training to offer a safe and realistic tactile learning approach [6].

Here, we wanted to identify currently available simulators, evaluate the evidence of their effectiveness, and assess their adoption within the neurosurgical community [102, 103]. By doing so, we review the nature of available simulator varieties with the aim of supporting neurosurgical educators and decision-makers in selecting the best simulation approach for their trainees.

## Methods

### Search strategy

**Fig. 1:**
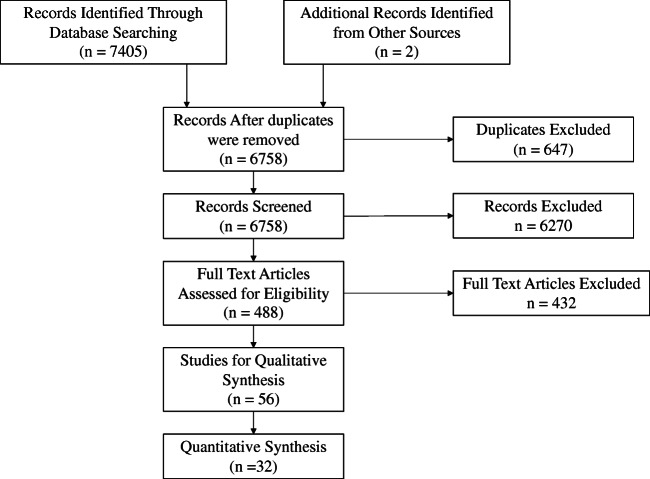
PRISMA flow chart of the systematic process utilised for the review. Fifty-six articles were included in the final qualitative review with 32 in a quantitative meta-analysis including 7 randomised studies measuring various outcome domains

The objective was to characterise and appraise the literature for outcomes associated with neurosurgical simulation education. The study was registered on PROSPERO (number CRD42019144840). A multiplatform database search was conducted with the terms “Neurosurgery, Simulation and Education” on the OVID platform including the following databases: Books@Ovid (July 19, 2019), Journals@Ovid Full Text (July 19, 2019), CAB Abstracts (1910 to 2019 week 28), Embase (1974 to present), GeoRef (1666 to July week 03 2019), Medline (Ovid MEDLINE Epub Ahead of Print, In-Process and Other Non-Indexed Citations, Ovid MEDLINE Daily and Ovid MEDLINE) (1946 to present), PsycINFO (1806 to present), Zoological Record Archive (1864 to 1977), Zoological Record (2009) (*Supplementary Table* *1*). Additionally, the PubMed platform was searched. An extended literature search for progressive scholarly acceptance used keywords “virtual reality/augmented reality and neurosurgery” in all databases including PUBMED, OVID-MEDLINE, HDAS, and SCOPUS. A PRISMA- and PICOS-guided selection of the imported results onto the Rayyan web platform was screened by two blinded independent resident researchers with expertise in neurosurgical education (JD, SM) (see PRISMA flowchart in Fig. 1) [80]. Included articles were imported into endnote reference manager (Clarivate Analytics *Version X.9.3.2*). HJM was the tie breaker resolving conflicts that arose during article selection post-screening.

### Eligibility criteria

Selected articles satisfying our inclusion criteria reported on primary simulation research, digital simulations including AR, VR, and mobile phone platform-based simulation. Articles were included if they were published in the English language and described simulation-based neurosurgical intervention used in a training setting for the acquisition of procedural knowledge or technical skills. We also included articles that presented patient outcome data following simulation training in neurosurgery, articles looking at cadaveric simulation models in neurosurgery, those describing microsurgical skills, and articles describing machine learning modelling methods in simulating neurosurgery with an educational component. We excluded publications discussing simulation with little or no reference to neurosurgery and education, or that focused exclusively on non-technical skills.

### Validity and bias assessment

The Medical Education Research Study Quality Instrument (MERSQI) checklist was used to quantify the validity of studies that reported on neurosurgical simulation education [20, 90]. MERSQI was also used to evaluate study quality and bias. The Cochrane risk of bias tool was additionally applied to RCTs with an assessment of its seven key components [47]. Disagreements regarding quality or bias assessments were resolved through discussion with a senior author (HJM).

### Meta-analysis

A meta-analysis was performed for cohort studies and randomized trials that identified improvement in procedural knowledge and technical skills as outcomes achieved using neurosurgical simulation. We used STATA (StataCorp. 2013. *Stata Statistical Software: Release 13*. College Station, TX: StataCorp LP.) for random effects modelling. Outcome measures for procedural knowledge were scores on assessments. Outcome measures for technical skills included accuracy; speed (time to task completion and speed of task completion); and other metrics (error, comfort, number of fluoroscopy shots used). For each outcome measure, meta-analyses were performed using all relevant data sources regardless of simulation protocol. A normalised ratio of means (*R* = [$\overline{X}$_E_-$\overline{X}$_C_]/$\overline{X}$_C_) analysis was adopted because of different outcome scales, where a mean difference or standardised mean difference effect estimate would have been inappropriate. A random effects model using an inverse variance DerSimonian Laird estimator was used for between-study variance with confidence intervals. Study heterogeneity was appraised through the *I*^2^ statistic. Significance was set at *p* < 0.05. The meta-analysis was reported using the QUORUM guidelines [74]. Authors were contacted for missing data. Incomplete sets of parameters were automatically excluded from the models without imputation of missing values.

### Progressive scholarly acceptance analysis

The progressive scholarly acceptance (PSA) metric was applied to assess the current stage of acceptance of VR simulation as an educational tool within the global neurosurgery community [102, 103]. In accordance with the original intention of the PSA metric, we defined ‘initial/ refining’ studies as follows: (i) initial studies generally only described simulation with no evidence of its use in training and demonstrated the development of VR simulation models for neurosurgical procedural planning or illustration of neurosurgical anatomy, but no direct evidence of their use for neurosurgical training, (ii) refining studies demonstrate the use of VR simulation models for the training of users in any aspect of neurosurgical practice with an objective or subjective assessment of skill acquisition. PSA is defined as the point in time at which the total number of refining studies exceeds the total number of initial studies, indicating community acceptance of the chosen intervention. The initial time point was defined as the year of publication of the first initial study identified.

## Results

### Search strategy

We screened 7405 article titles and abstracts, removed 647 duplicates, and excluded 6758. Of the 488 full-texts, 56 studies were included in the final review of which 32 studies including 7 randomised clinical studies were also included in the meta-analysis. The PRISMA flow chart summarises the review parameters and results (Fig. 1). Tables 1, 2, and 3 summarise search methodology and the included studies. Additional detailed data tables are summarised in *Supplementary Tables* *1**and* *2*.

**Table 1:** The search strategy of this systematic review performed on the electronic databases of OVID MEDLINE, PUBMED, EMBASE, and PsycINFO. The table indicates the stages of the search strategy. Stage 1 shows areas of simulation, stage 2 searched for areas on education, and stage 3 searched for neurosurgery. The searches were then combined in stage 4

1.) (simulat* OR comput* OR model* OR technolog* OR tactile OR haptic* OR robot* OR ‘augmented reality’ OR ‘virtual reality’ OR ‘artificial intelligence’ OR ‘animal model*’).mp. [mp = tx, bt, ti, ab, ct, sh, ot, hw, id, cc, tn, dm, mf, dv, kw, fx, dq, ba, bk, ca, cl, cw, yr, nm, kf, ox, px, rx, an, ui, ds, on, sy, tc, tm, mh] 2.) (educat* OR train* OR teach* OR learn* OR curricul* OR competen* OR skill*).mp. [mp = tx, bt, ti, ab, ct, sh, ot, hw, id, cc, tn, dm, mf, dv, kw, fx, dq, ba, bk, ca, cl, cw, yr, nm, kf, ox, px, rx, an, ui, ds, on, sy, tc, tm, mh] 3.) (neurosurg* OR neuro-surg*).mp. [mp = tx, bt, ti, ab, ct, sh, ot, hw, id, cc, tn, dm, mf, dv, kw, fx, dq, ba, bk, ca, cl, cw, yr, nm, kf, ox, px, rx, an, ui, ds, on, sy, tc, tm, mh] 4.) 1 AND 2 AND 3

**Table 2:** The number of selected studies based on neurosurgical subspecialty for which simulation was targeted. Limited to studies performed between 2011 and 2019. Spinal neurosurgery categorises the studies published referencing neurosurgery and education in peer-reviewed neurosurgical journals

Neurosurgical area of simulation	Number of selected articles
Cranial neurosurgery total	36
Cranial neurosurgery: functional	1
Cranial neurosurgery: hydrocephalus	9
Cranial neurosurgery: neuro oncology	3
Cranial neurosurgery: neurovascular	14
Cranial neurosurgery: unspecified	6
Cranial neurosurgery: skull base and pituitary	3
Unspecified subspecialty general neurosurgery total	5
Spinal neurosurgery total	14
Spinal neurosurgery: paediatric	1
Spinal neurosurgery: adult	13
Spinal surgery total	1
Grand total	56

**Table 3:** Studies that were performed between 2011 and 2019 across different continents. This data shows the number of selected articles by country of publication. Higher proportions of identified studies originated from the USA, followed by China, UK, Canada, and Italy

Geographical location	Included publications
USA	39
UK	5
Italy	2
China	5
Canada	5
Grand total	56

### Systematic review

The selected studies performed from 2011 to 2019 that discuss educational simulation in neurosurgery are summarised in *Supplementary Tables* *1**and* *2*. Fifty simulator types were identified. Since 2011, simulation studies reported from the USA accounted for 69.6% (*n* = 39), 7.6 times greater than China and Europe with each accounting for 8.9% (*n* = 5) of the total selected. Of the percentage of studies selected, 64.3% (*n* = 36) had a documented focus on cranial neurosurgical simulation methods compared with 25% (*n* = 14) being focused on spinal procedures. Twenty-five percent of the total selection (*n* = 56) accounting for a 38.9% (*n* = 14) of the cranial neurosurgical simulation studies were neurovascularly focused, 3.6% were related to skull-base neurosurgical procedures (*n* = 2), significantly lower than 16.1% (*n* = 9) that focused on hydrocephalus-related disorder management. However, pituitary (1.8%, *n* = 1), functional (1.8%, *n* = 1), and paediatric spinal surgical (1.8%, *n* = 1) simulations had the lowest documented representations. For our timeframe of focus (early 2011 till present), we identified a peak in neurosurgical simulation innovation in 2013 with 25% (*n* = 14) compared with 17.9% in 2018 discussing new devices for various outcome measures in simulation.

### Meta-analysis

Our meta-analysis of 32 authored studies followed assumptions considering whether trainees benefitted through simulated skills improvement for training under standardised conditions. Accordingly, we sought to determine whether studies supported bench-to-bedside translation for simulation in clinical neurosurgical settings by augmenting trainee experience which in turn suggests improved patient outcomes. Normalised outlier means exceeding a value of 1 were assumed to have a value close to one to avoid omission and selection bias against these studies.

#### Procedural Knowledge

Twenty-one studies were included (*N* = 55 sub-studies). A significant 82.7% improvement in knowledge in all outcome domains was demonstrated (ES 0.827, CI, 0.820–0.833, *p* = 0.0001). Additionally, a significant improvement of 50% (ES 0.502, CI 0.355; 0.649, *p* < 0.001) was seen using simulation in the context of objective structured assessments to facilitate procedural knowledge acquisition. The highest effect size estimate with an improvement of up to 99% (ES 0.999, CI 0.997–1.001, *p* < .0.001) was seen for objective score-based simulation methods for knowledge acquisition (see Fig. 2a–c).

**Figure:**
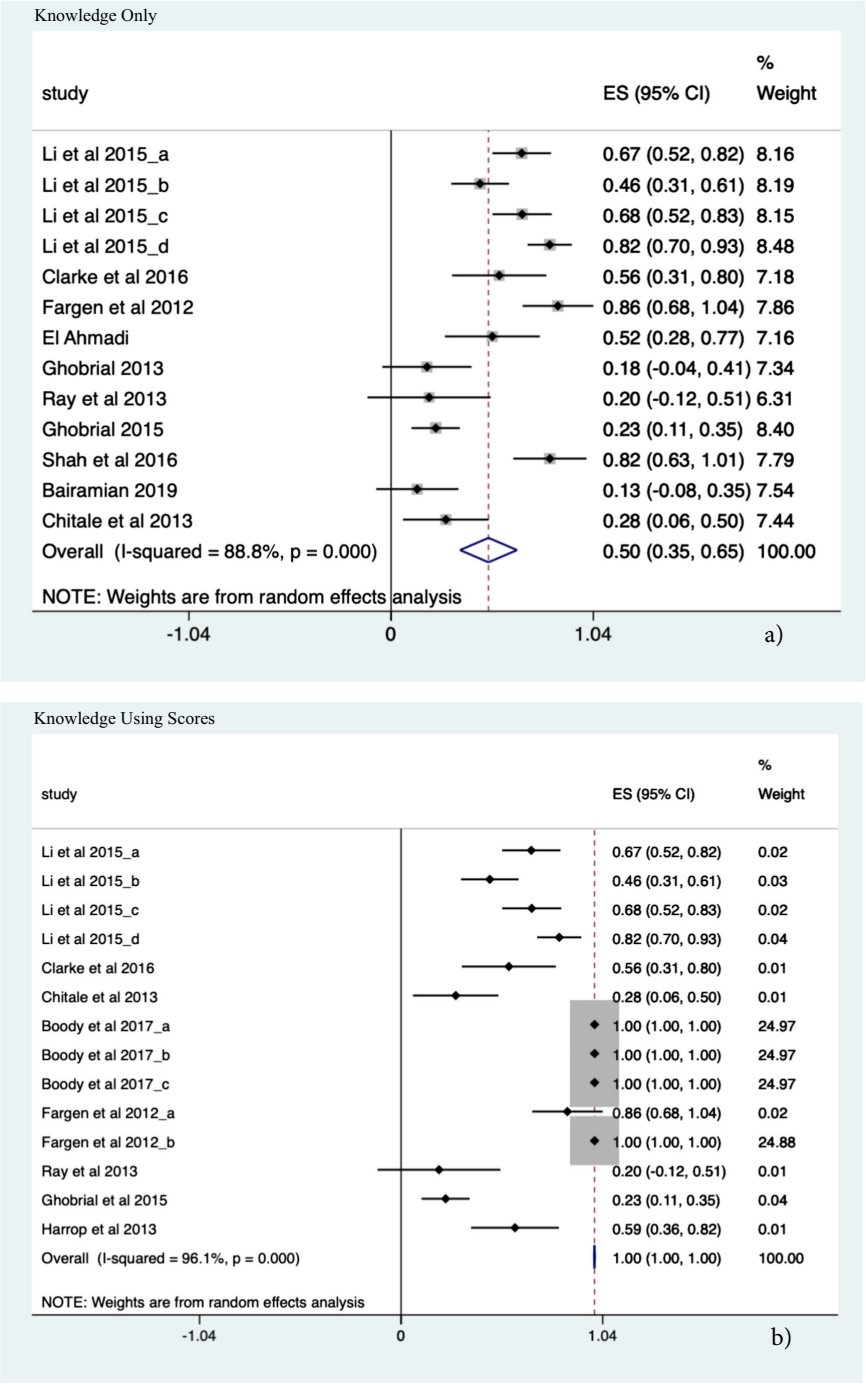

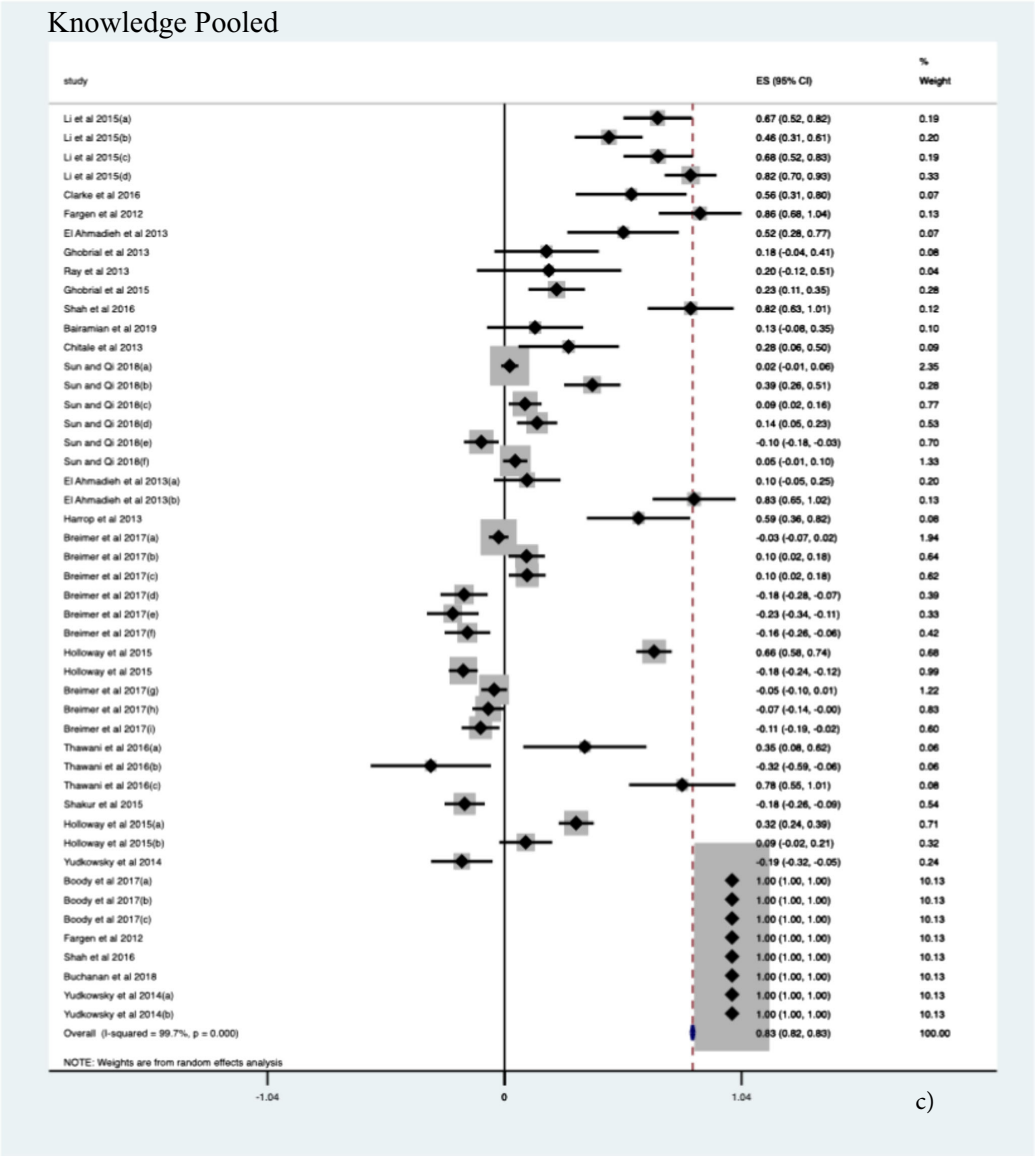

#### Technical skill

##### Accuracy

**Fig. 3:**
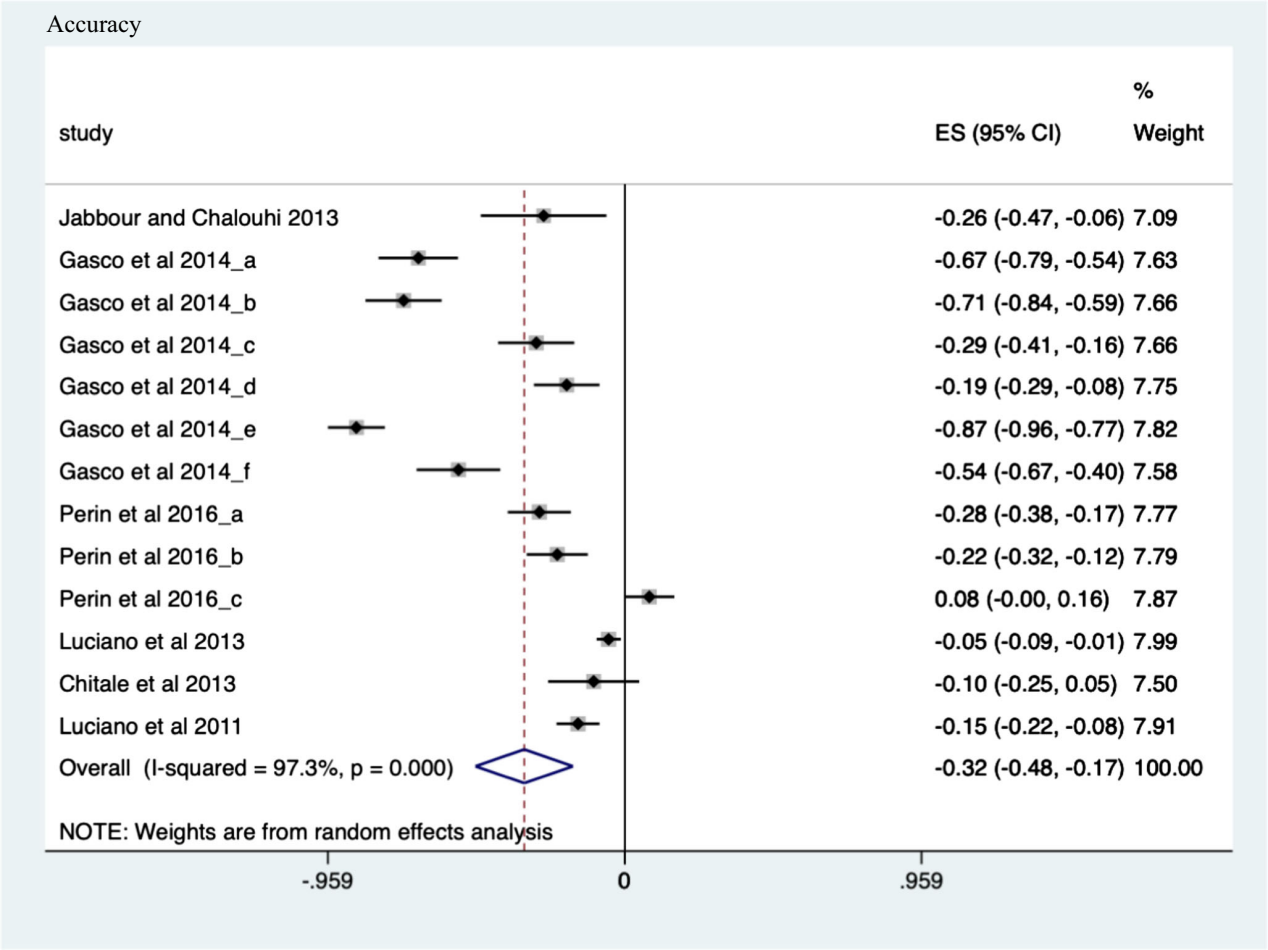
Accuracy meta-analysis. Forest plot of pooled studies investigating outcome measure of accuracy. Supplementary data summarises the measures calculated for this domain in simulation and meta-analysis results of measured improvement in accuracy. A random effects model as shown. Statistical significance set at *p* < 0.05

Of the 6 included studies (*n* = 13 sub-studies), statistical heterogeneity in the ratio of means outcome measure of accuracy was noted (*I*^2^, 97.3%). However, a significant improvement of 32.5% (ES 0.325, CI, − 0.482; − 0.167, *p* < 0.001) accuracy in all learners’ acquisition of procedural skill using simulation was noted (see Fig. 3).

##### Speed

Ten studies were included in the final meta-analysis (*N* = 21 sub-studies). A significant effect size was demonstrated for pooled ratio of means assessment of speed (ES 0.427, CI − 0.403; 0.450, *p* = 0.0006 (see Fig. 4). Additionally, procedural-specific speed improvements measured as a ratio of time to task completion across multiple domains was a factor of 3.95 times faster using various forms of simulation. An absolute effect size estimate of 0.253 (ES − 0.253, CI − 0.399; − 0.107, *p* < 0.001) is seen.

**Figure:**
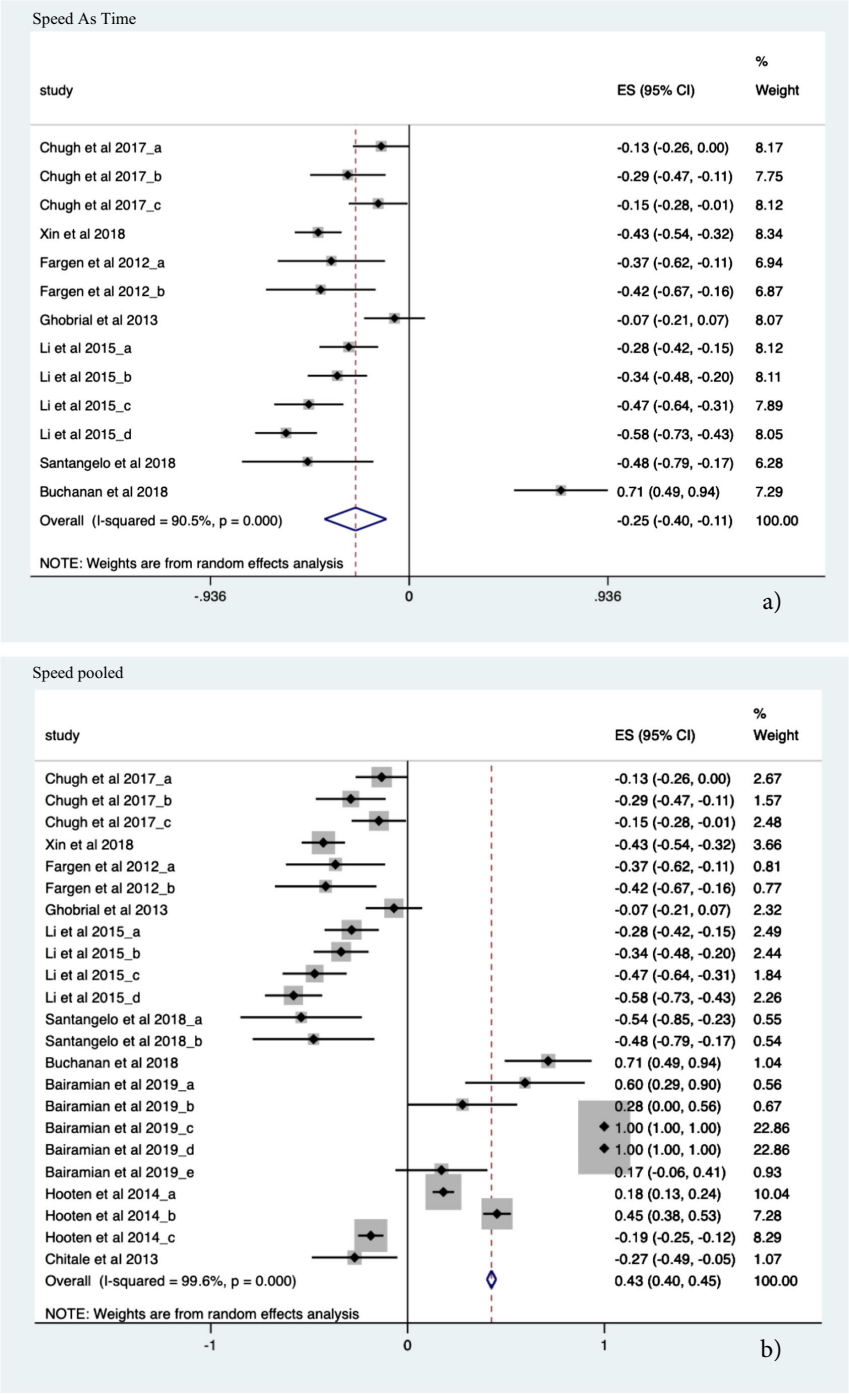

##### Other metrics

More objective procedural-related safety parameters included three studies (*N* = 6 sub-studies) related to measuring the depth of perception, mean distance from target tissue, and consistency of forces applied showing a significant 21.2% improvement (ES − 0.212, CI − 0.307; − 0.116; *p* < 0.001) in these areas (*Supplementary Figure* 1).

Seven studies (*n* = 18 sub-studies) were included in the meta-analysis and showed no significant improvement across the domains of procedure-related comfort (ES − 0.013, CI − 0.411; − 0.386, *p* = 0.951) and administered fluoroscopy shots (ES − 0.082, CI − 0.179; 0.016, *p* = 0.102) (*Supplementary Figure* *2*).

The average MERSQI was 11.5 ± 2.2 (SD, 95% confidence level of 0.626). The Cochrane risk of bias assessment is summarised in (*Supplementary Figure* 3)*.*

##### Progressive scholarly acceptance

The PSA metric indicates that the use of simulation technologies like VR for neurosurgical simulation has not reached ‘progressive scholarly acceptance’. The initial study by Auer and Auer identified in the literature search was published in 1998, which was set as the initial time point [3]. Over a 20-year period (1998–2018), the number of initial studies (*n* = 91) was approximately double the number of refining studies (*n* = 45) (*Supplementary Figure* 4*b*). However, the latter decade (2008–2018) demonstrates a surge in the number of refining studies, with consistently increased numbers of studies compared with initial studies between 2013 and 2017 (28 vs 22 studies) (*Supplementary Figure* 4*a*).

## Discussion

### Principal findings

Technological developments have stimulated a growing interest in using simulation for neurosurgical training over the past decade. In this review, 50 simulator subtypes ranging from cadaveric, low-fidelity, and part-task to VR simulators were identified. Collectively, the use of these simulators was associated with a significant 82.7% (ES 0.827, CI, 0.820–0.833, *p* = 0.0001) improvement in procedural knowledge in all outcome domains. The measurement of technical skills was based on procedure-specific speed improvement as a ratio of time to task completion across multiple domains. This was 3.95 times faster using various forms of simulation (ES − 0.253, CI − 0.399; − 0.107, *p* < 0.001). Additionally, there was a significant improvement of 32.5% accuracy in all learners’ acquisition of procedural skills using simulation (ES 0.325, CI, − 0.482; − 0.167, *p* < 0.001). The use of VR studies has increased in recent years. However, our bibliometric analysis suggested we are yet to reach widespread acceptance of VR simulation as an integral part of neurosurgical training. The number of initial studies (*n* = 91) was approximately double the number of refining studies (*n* = 45) over a 20-year period, although 2008–2018 showed a surge in the number of refining studies compared with initial studies between 2013 and 2017 (28 vs 22 studies).

### Comparison with other studies

Although cadaveric methods of neurosurgical simulation education had long been the gold standard method for neurosurgical education, there has been a slow shift towards digital methods in recent decades [4, 9, 13, 16, 27, 29–31, 39, 44, 51, 54, 58, 63, 65–68, 72, 81, 98, 99, 104, 111, 114, 117, 122]. Our review identified that 5.36% of cadaveric studies were used in association with digital technologies like VR to augment neurosurgical learning [51, 115, 121]. A disadvantage of cadaveric approaches alone is the inability to simulate dynamic pathology or complications such as bleeding or CSF leak. Other concerns regarding cadaveric use include unknown disease vector transfer to trainees, ethico-legal uncertainties, and infrastructural expense for cadaver storage [105]. By contrast, current achievements in graphics technologies have enabled virtual/augmented, mixed reality, and 3D printed technologies for better simulation learning experiences with improved photorealistic fidelity [1, 4, 5, 7–9, 12, 13, 15–17, 19, 24, 25, 27, 29, 30, 32, 34, 35, 39, 44, 48, 50, 51, 54, 58, 60, 62, 63, 65, 68, 70, 72, 81, 82, 88, 97–101, 104, 106, 111, 114, 116, 117, 122, 124, 125].

From our meta-analysis, we hypothesised whether trainees benefitted through simulated skills improvement for training under standardised conditions. Accordingly, we sought to determine whether studies supported bench-to-bedside translation for simulation in clinical neurosurgical settings by augmenting trainee experience, which in turn improves patient outcomes.

A non-significant improvement in safety through minimising errors associated with procedural-related discomfort was identified, whereas objective procedural-related safety parameters such as depth perception and minimisation of tissue injury showed significant improvement and thus warrant further study. The high degree of heterogeneity is linked to inadequate standardisation and culturally distinct methods of international surgical practices. This in turn influences procedural simulation study design. Moreover, whilst certain institutions may be early adopters with exposure to digital neurosurgical simulation technologies, others may be unable to gain the required exposure due to organisational financial constraints. In fact, various teams have highlighted that the costs of neurosurgical simulation can be high and hence have developed further methods like app-based communication platforms to reduce costs [26, 28, 31, 61, 75, 79, 113]. Study designs for centres with established track records and budgets for neurosurgical simulation differ from centres with financial constraints undertaking an initial study into its usefulness for their trainees. This temporal experiential discrepancy between early and late adopters of digital neurosurgical simulation technologies could limit study designs and bring about design-related heterogeneity.

There is also clinical baseline heterogeneity where within-study participants vary. Some studies had neurosurgical simulation implemented using both students (novices) and graduate doctors (experts and intermediates) at varying levels of their practice, hence different baseline characteristics, some in an assessment capacity [8, 27, 35, 48, 51, 58, 72, 82, 101, 110, 124]. Similar statistical heterogeneity occurred in the ratio of means outcome measure of accuracy.

Evidently, as newer simulation technologies appear and gain traction for use in various subspecialties, results may not generalise across all domains or translate to assessing impacts on most patient clinical outcomes [5]. As most studies have been based in the USA to date, it remains to be seen whether the results are skewed by the cultural norms of a country. It also remains to be assessed whether confounders such as cultural pre-framing of the participants in the simulation process, the problem to be solved, and the length of time required to solve it all influence the cross-border standardisation of neurosurgical simulations. This becomes worthy of exploring in future studies in a standardised environment for policymakers. Moreover, the risk of publication bias is associated with the challenges in blinding, which may also have contributed to sample heterogeneity (see *Supplementary Figure* 3). Rhodes and colleagues report that up to 37% (95% interval: 0–71%) heterogeneity variance could be explained by trials at high/unclear risk of bias [96]. The average MERSQI was 11.52 ± 2.20 (SD, 95% confidence level of 0.626) suggesting a 25.0% improvement from a decade ago reported by Kirkman and colleagues 9.21 ± 1.95 (SD; range 6–12.5) [56]. The majority of the selected prospective randomised controlled trials were single-blinded trials, as double-blinding to reduce the risk of bias appears technically challenging in simulation trials with only one achieving this [122].

One randomised controlled trial (RCT) looked at VR simulation on patient-reported outcomes of efficacy that also offered a patient-related educational slant on perioperative care delivery and its effect on patient outcome [5]. Considering the paucity of quality improvement initiatives, Bekelis and colleagues performed a randomised clinical trial of patients undergoing cranial and spinal surgical procedures evaluating the use of an immersive pre-operative VR set-up compared with operation-type stratified standard pre-operative experience. Outcomes measured included the Evaluation du Vecu de l'Anesthesie Générale (EVANG) and Amsterdam Preoperative Anxiety and Information scoring systems (APAIS) gauging patient perioperative satisfaction. They reported an improved EVANG and high APAIS score (difference, 29.9; 95% CI, 24.5–35.2) together with lower patient stress scores (VAS; difference of − 41.7; 95% CI, − 33.1 to − 50.2) with patients feeling better prepared (difference, 32.4; 95% CI, 24.9–39.8) for their procedures in the pre-operative period and no association of VR simulation with VAS stress score.

Progressive scholarly acceptance (PSA) which demonstrates an appreciation for how the scientific community accepts emerging technologies in VR-based neurosurgical simulation has not yet been undertaken to the authors’ knowledge [102, 103]. We performed a progressive scholarly acceptance review on VR models in neurosurgery. The PSA results support that compounded initial studies using VR are still exponentially increasing as new types of simulators are frequently being introduced to facilitate and augment neurosurgical education [102, 103]. Evidently, there is a clear divergence between compounded initial studies and refining studies for scholarly acceptance to be reached. On the contrary, the individual publications each year seem to suggest that there is a dual crossover of the initial and refining studies in 2013 and 2017. The year 2013 was identified in our analysis as the modal year for published studies on neurosurgical simulation (*Supplementary Figure* 4*A and B*).

PSA analysis suggests that we are yet to reach the point of widespread acceptance of VR simulation as an integral part of neurosurgical training. However, a sustained increase in the annual number of refining studies over the last decade suggests that we will soon see ‘progressive scholarly acceptance’. Whilst the PSA provides an innovative attempt at capturing the difficult concept of community acceptance for a given simulation intervention, it is not without limitations as outlined in its seminal publication [102, 103].

### Limitations

Our meta-analysis was conducted on a heterogeneous dataset. Nonetheless, it must be appreciated that studies will have been conducted at different institutions without an internationally standardised methodology, because of various neurosurgical simulations being such early-stage technologies. As this is a rapidly evolving field, extrapolating significant results to clinical practice should be considered with caution. We are at a stage where a global multi-centre randomised controlled crossover study for a single improvement domain for neurosurgical education would be warranted in effectively guiding clinical practice.

The main limitation of our PSA analysis lies with the definition of initial and refining studies, which may be difficult to distinguish at times. We defined initial studies as demonstrating the use of VR simulation models for neurosurgical procedural planning or illustration of neurosurgical anatomy, but not direct evidence of use in neurosurgical training. However, one may argue that this definition is not specific to neurosurgical training given that these VR models could be used for pre-procedural planning alone for fully trained neurosurgeons. This reduced stringency for the inclusion of initial studies may have resulted in a disproportionately greater number of initial studies compared to refining studies. Consequently, this would reduce the likelihood of the PSA metric indicating widespread acceptance of VR simulation for education by the neurosurgical community. Nonetheless, we agreed that this was the optimal definition for initial studies as even the use of VR simulation for pre-procedural planning alone should ultimately culminate in improved performance of fully trained neurosurgeons as well, which is a key element of education and training. Essentially, whilst VR simulation is rapidly gaining attention for its potential role in neurosurgical education, we are in dire need of further studies illustrating objective improvement to further establish its role within the neurosurgical curriculum.

### Future directions

An important area that was not fully appraised or discussed by the selected studies involved the psychological aspects of education training delivery using a debriefing process. As the learner reflects during the debrief, it is considered the most important period where enhanced learning experience is achieved. To our knowledge, only a handful of studies in our series made subtle attempts at reporting on the debriefing process, when it comes to neurosurgical simulation education. There is no established consensus on whether the debrief period for digital technological-based simulations designed for neurosurgical training purposes should differ from current methods. Currently, the usual duration for debriefing outside the virtual reality environment (exo-virtual debrief) is 2 to 3*x*, where *x* is the duration of time for the simulation activity. With training time constraints and the need for accelerated service delivery, extended debriefing may not be feasible.

Moreover, in attempting to circumvent these potentially negative impacts of the process, will an exo-virtual debrief be a requisite component of the total debrief period in order to manage subjective detachments from reality noted to be linked to post-VR autonomic dysregulation that occurs with inter-individual variability [10, 78]? Consequently, such novel methods could facilitate in-VR-to-reality reorganisation therapy of the senses after simulation experiences and may be effective either conducted together with traditional debriefing methods or alone. It is reasonable to consider if this may also be like the traditional duration (amounting to the 2–3*x* period) or whether a shortened debrief process should be determined.

Digitisation and automated pooled video analytics of procedures from laypeople are starting to gain recognition and have an advantage of being fast, objective, and cheaper than an expert [49, 59]. Currently, global educators are leveraging internet platform-based technologies to deliver neurosurgical operative education across continents that have little access to technical expertise thereby rapidly bridging the knowledge gap [36, 55, 77, 87].

When it comes to cognitive and social congruences, evidence is linked to distance along the near-peer simulation training spectrum [42]. One study in the neuroanatomical educational environment showed that cognitive and social congruence is influenced by distance along the near-peer teaching spectrum [42], although such phenomena are yet to be fully appraised in VR- and AR-based educational simulation environments.

The impact of artificial intelligence especially neural networks in enabling future objective procedural knowledge and skills analysis as well as for tele-neurosurgery requires further mention [52, 53, 89, 107]. Techniques such as Hidden Markov Models, Support Vector Machines, and other deep learning methods like convolutional neural networks offer tremendous potential for automated feedback directed learning [45, 92–95, 112]. Further clinical studies will be necessary for face, content and construct validity of these techniques for automated feedback-driven advanced neurosurgical procedural knowledge and skills training. Telesurgery in robotic endonasal surgery extends concepts of procedural practice and training over long distances. Wirz et al. demonstrated that phantom pituitary tumour removal can be performed by a surgeon controlling a robot located approximately 800 km away [108, 120]. In combination with platform procedural tele-mentoring, newer avenues of enhanced procedural feedback training could be delivered.

## Conclusions

Operative neurosurgery will continue to benefit from the currently evolving simulation technological revolution for education. Accordingly, there is strong evidence for a beneficial effect of simulation in the improvement of accuracy, time to completion of procedural tasks, and knowledge; however, the size of the effect is yet unclear. We show that areas such as virtual reality in neurosurgical educational practice may not have yet or only partially gained progressive scholarly acceptance. Nonetheless, an understanding of whether other simulation technologies will become completely accepted in practice within the surgical community remains to be fully appreciated as further time-dependent evaluative studies become necessary to reach full progressive scholarly acceptance. Cumulative work will allow the occurrence of progressive scholarly acceptance soon, but robust study designs with consensus standardised metrics will become imperative in order to achieve this.

## Electronic supplementary material

ESM 1 Supplementary Table 1 Studies published between 2010 till present since the last published review by Kirkman et al.[56] Studies classed as RCT = Randomised Controlled Trials had evidence of blinding and randomisation, authors specifically referring to their studies as such. Studies that did not describe blinding but only randomisation were classified as Randomised studies. Average calculated MERSQI was 11.52 ± 2.20 (Mean, SD), Range 5-15. [1, 2, 4, 5, 7–9, 12, 13, 15–17, 19, 23–25, 27, 29–32, 34, 35, 39, 43, 44, 48, 50, 51, 54, 58, 60, 62–70, 72, 81–83, 88, 97–101, 104, 106, 110, 111, 114, 116, 117, 122, 124, 125]. Supplementary Table 2 Clinical trials included in the final quantitative meta-analysis were analysed based on improvement in a particular domain. Ne is the sample number of the intervention/event outcome measure, Nc is the sample number of the control measure or comparator, Se is the standard deviation of the sample outcome mean, Sc is the standard deviation of the comparator mean. Me is the mean of the sample outcome, Mc is the mean of the comparator. P-value is set at 0.05. (DOCX 194 kb)

ESM 2 (PDF 294 kb)
