# Supplementary material for: Simulation for skills training in neurosurgery: a systematic review, meta-analysis, and analysis of progressive scholarly acceptance
Source: Neurosurg Rev. 2020 Sep 18;44(4):1853–67. doi: 10.1007/s10143-020-01378-0 (PMC8338820; doi:10.1007/s10143-020-01378-0)
Supplement: Supplementary file 1 — Supplementary Table 1 Studies published between 2010 till present since the last published review by Kirkman et al.[56] Studies classed as RCT = Randomised Controlled Trials had evidence of blinding and randomisation, authors specifically referring to their studies as such. Studies that did not describe blinding but only randomisation were classified as Randomised studies. Average calculated MERSQI was 11.52 ± 2.20 (Mean, SD), Range 5-15. [1, 2, 4, 5, 7–9, 12, 13, 15–17, 19, 23–25, 27, 29–32, 34, 35, 39, 43, 44, 48, 50, 51, 54, 58, 60, 62–70, 72, 81–83, 88, 97–101, 104, 106, 110, 111, 114, 116, 117, 122, 124, 125]. Supplementary Table 2 Clinical trials included in the final quantitative meta-analysis were analysed based on improvement in a particular domain. Ne is the sample number of the intervention/event outcome measure, Nc is the sample number of the control measure or comparator, Se is the standard deviation of the sample outcome mean, Sc is the standard deviation of the comparator mean. Me is the mean of the sample outcome, Mc is the mean of the comparator. P-value is set at 0.05. (DOCX 194 kb) [file 10143_2020_1378_MOESM1_ESM.docx]

| **Authors** | **Year** | **Location** | **Study Design** | **Simulator Discussed** | **Measurement of Outcomes** | **P-Value** | **Neurosurgical Area of Simulation** | **MERSQI** | **Ref** |
| --- | --- | --- | --- | --- | --- | --- | --- | --- | --- |
| Luciano et al 2011 | 2011 | USA | Cohort Study Experimental/Assessment based | High-resolution augmented reality simulator with haptic feedback | A study to assess learning retention of thoracic pedicle screw placement using a high-resolution augmented reality simulator with haptic feedback in Fellows and Residents, Showing a trend in improvement | p<0.08 | Spinal Neurosurgery | 13 | [63] |
| Fargen et al 2012 | 2012 | USA | Cohort Study: Assessment-based | Vist-C Simulator Systems | Pre and Post Course written assessments showing that Post-course written test scores were significantly higher than pre-course scores. Faculty assessments of participants’ technical skills with angiography (graded 0e10, with 10 being best). Objective simulator recorded assessments demonstrated a significant decrease in the time needed to complete a four-vessel angiogram (p<0.001) and total fluoroscopic time (p<0.001). | p<0.001 | Cranial Neurosurgery: Neurovascular | 14 | [25] |
| Lau et al 2012 | 2012 | USA | Experimental Video-Based Design | Video | Video simulation to demonstrate safety within an operative neurosurgical environment. |  | General Neurosurgery | 5 | [59] |
| Ganju et al 2013 | 2013 | USA | Cohort Study: Survey Based Design | Survey Questionairre | 14-item questionnaire was emailed to 99 Neurosurgery residency program directors to assess the clinical impact of simulation, response rate was approximately 54% within 2 weeks, Cost analysis |  | General Neurosurgery | 9.5 | [27] |
| Choudhury et al 2013 | 2013 | CANADA | Cohort Study: Survey Based Design | NeuroTouch Virtual Reality | NeuroTouch Virtual Reality Simulator integrated with standardisation of training via Fundamentals of neurosurgical training, survey and expert interviews. |  | Cranial Neurosurgery: Pituitary, Neurooncology, Hydrocephalus, Neurovascular | 12 | [16] |
| El Ahmadieh et al 2013 | 2013 | USA | Cohort Study: Survey Based Design | Objective Microanastomosis Assessment Tool Scale | Comparison of cognitive and technical performances using Didactic, objective structured assessment of technical skill (OSATS), Northwestern Objective Microanastomosis Assessment Tool Scale, operative microscope, a full microsurgical kit, two 3-mm synthetic vessels, 8-0 nylon sutures, and a pump |  | Cranial Neurosurgery: Neurovascular | 13 | [24] |
| Gasco et al 2013 | 2013 | USA | Cohort Study: Survey Based Design | ImmersiveTouch simulation platform | ImmersiveTouch simulation platform, Novel Virtual Reality Simulation for Hemostasis |  | Cranial Neurosurgery: Neurovascular | 10 | [29] |
| Gasco et al 2013 | 2013 | USA | Cohort Study: Survey Based Design | Likert Scale Evaluation | Cost Analysis, Global Rating Scales of Operative Performance to study correlation of self-perceived (sub- jective) and faculty-perceived (objective) levels in the Physician Performance Diagnostic Inventory Scale (PPDIS) for a selected group of procedures performed at the end of the curriculum schedule | p<0.001 | General Neurosurgery | 12.5 | [31] |
| Ghobrial et al 2013 | 2013 | USA | Cohort Study: Assessment-based | Sawbones laminectomized spine model Simulator, Standard Durotomy Repair Model, Questionairre | sawbone reproduction of the lumbar spine (standard durotomy repair model), 11-question written pretest to assess current knowledge of relevant spinal anatomy, knowledge of CSF repair techniques, Didactic and Technical based educational models, pre- and posttest scores of 1.17 (18.5%; P = .02) whereby the median leak rate improved from 14 to 7 (P = .34). There were also demonstrative technical improvements by all. | p<0.02 | Spinal Neurosurgery | 10 | [34] |
| Harrop et al 2013 | 2013 | USA | Cohort Study: Assessment-based | Posterior cervical decompression spinal simulator | posterior cervical decompression simulator, with didactic scoring of 5 postgraduate year (PGY) 2 participants, 1 PGY-3 participant, 2 PGY-5 participants and 1 attending, objective structured assessment of technical skill | P = .005 | Spinal Neurosurgery | 12 | [44] |
| Jabbour and Chalouhi 2013 | 2013 | USA | Cohort Study: Assessment-based | Stryker pre-sigmoid approach simulation module | Stryker pre-sigmoid approach simulation module; mean number of hits to the dura, facial nerve, and sigmoid sinus decreased from 4.2 in the first test to 3.1 in the second test | P <.05 | Cranial Neurosurgery: Skull base | 11 | [54] |
| Mattei et al 2013 | 2013 | USA | Experimental Pilot Study | NextEngine 3D computer simulator, 3D scanner | 3D Scanner HD, NextEngine, Inc of the lumbar vertebrae, 3D computer simulation of the relationship of the tissues involved in open neural tube defects |  | Spinal Neurosurgery: Paediatric | 11.5 | [72] |
| Marcus et al 2013 | 2013 | UK | Preclinical Study | MARTYN | The use of physical models, such as the Modelled Anatomical Replica for Training Young Neurosurgeons (MARTYN) that has been developed by the conservation team at the RCS, allows standardisation, reproducibility and the ability to incorporate pathology in a manner that animal models and cadavers rarely can |  | Cranial Neurosurgery |  | [66] |
| Ray et al 2013 | 2013 | USA | Cohort Study: Assessment-based | Anterior Cervical Discectomy Spinal Simulator | anterior cervical discectomy and fusion (ACDF) simulator |  | Spinal Neurosurgery | 13 | [88] |
| Schirmer et al 2013 | 2013 | USA | Cohort Study Experimental/Assessment based | ImmersiveTouch VR-based ventriculostomy simulator | A study conducted to enhance resident trainee to manage cerebrospinal fluid disorders incorporating VR simulators to help train residents in the management of common neurosurgical disorders, Improvement in procedural time completion, Senior residents planning better trajectories than junior residents. | P < 0.004 | Cranial Neurosurgery: Hydrocephalus, ICP | 13 | [101] |
| Luciano et al 2013 | 2013 | USA | Experimental/Assessment based | Part-task simulator | Evaluation of a part-task simulator with 3-dimensional and haptic feedback as a training tool for percutaneous spinal needle placement, improvement in performance score | P = 0.04 | Spinal Neurosurgery | 12 | [29] |
| Chitale et al 2013 | 2013 | USA | Experimental/Assessment based | Low fidelity simulation Pre and Post didactic test scoring | Study to confirm the importance of establishing an educational curriculum for teaching minimally invasive techniques of pedicle screw placement using a computer-enhanced physical model of percutaneous pedicle screw placement with simultaneous didactic and technical components. Low fidelity simulation Pre and Post didactic test scoring methodology on anatomy, pathophysiology, and technical aspects associated with image-guided pedicle screw placement. Non-significant trends in improvement across areas such as Mean fluoroscopy time also improved from 193 ± 161 to 141 ± 104 seconds per screw. | p<0.4 | Spinal Neurosurgery | 12 | [15] |
| Gasco et al 2014 | 2014 | USA | Randomised Study | Sawbones Spinal model simulator | usefulness of virtual simulation training for learning to place pedicle screws in the lumbar spine | p<0.0067 | Spinal Neurosurgery | 13.5 | [30] |
| Kshettry et al 2014 | 2014 | USA | Survey Based Design | Low Fidelity Survey Questionnaire | A survey of methods of Laboratory based Simulation across centres in USA, Non-validated. |  | General Neurosurgery | 9.5 | [58] |
| Patel et al 2014 | 2014 | USA | Randomised Study | ImmersiveTouch virtual simulator haptic-based | Determining if a computer-based simulation with haptic technology can help surgical trainees improve tactile discrimination using surgical instruments, Differentiation of firmness, Virtual Simulation using ImmersiveTouch simulator, synthetic brain cavity model consisted of urethane foam, Simulation training vs no simulation training. Simulation improved localisation of brain cavity structures P  =  0·0183, | p = 0·0032 | Cranial Neurosurgery: General | 13.5 | [81] |
| Hooten et al 2014 | 2014 | USA | Cohort Study: Experimental/Survey Based Design | Ventriculostomy simulator | Validation of a ventriculostomy simulator as a necessary training tool in neurosurgical residency, more experienced residents have statistically significant better scores and completed the procedure in less time, Experience was stratified according to postgraduate year (PGY). Improvement in average time by experience and average score | p = 0.003 | Cranial Neurosurgery: Hydrocephalus, ICP | 12 | [50] |
| Gelinas-Phaneuf et al 2014 | 2014 | CANADA | Cohort Study: Experimental/Survey Based Design | NeuroTouch is a virtual reality (VR) simulator | Study of construct validity of NeuroTouch is a virtual reality (VR) simulator developed for neurosurgical skill training, Participants completed the internal resection of a simulated convexity meningioma, Performance metrics included volume of tissues removed, tool path lengths, duration of excessive forces applied and efficient use of the aspirator, significant tumour removal between students and residents favouring senior residents. | p=0.002 | Cranial Neurosurgery: Neurooncology | 11.5 | [32] |
| Yudkowsky et al 2014 | 2014 | USA | Cohort Study: Experimental /Survey/Image Based Design | ImmersiveTouch system, a head- and hand-tracked augmented reality and haptic simulator | Patient CT brain library was used to create 15 virtual brains for the ImmersiveTouch system, a head- and hand-tracked augmented reality and haptic simulator validated by Neurosurgical residents, Increase in Post intervention Simulator cannulation success rates, and live procedure outcomes showed improvement in the rate of successful cannulation on the first pass. increased post-intervention incidence of deeper, contralateral (simulator) and third-ventricle (live) placements. |  | Cranial Neurosurgery: Hydrocephalus, ICP | 13 | [124] |
| Marcus et al 2014 | 2014 | UK | Cohort Study: Anatomical Model and Video-based Study | MARTYN and VisionSense | Modelled Anatomical Replica for Training Young Neurosurgeons (MARTYN) for Vascular and Neuro-endoscopic (VisionSense cases). Primary outcomes were Face and Content Validity, Time to Task Completion between Novices and Intermediate Experts | p=0.0047 | Cranial Neurosurgery: Neurovascular |  | [64] |
| Marcus et al 2015 | 2015 | UK | Cohort Study: Experimental Preclinical Randomised study | Modelled Anatomical Replica for Training Young Neurosurgeons (MARTYN) and VisionSense III neuroendoscopy | Use of Augmented reality and 3D-Printed Skull model anatomical replica used to assess 50 novices. Primary outcomes were, the time to task completion and tool path length which differed significantly among the study groups | P = 0.002 | Cranial Neurosurgery: Neurovascular |  | [68] |
| Ghobrial et al 2015 | 2015 | USA | Cohort Study: Assessment-based | Cervical Laminoforaminotomy, OSAT, Educational Performance | cervical laminoforaminotomy and durotomy repair module, Obstructive Structured Assessment Test (OSAT) Performance scoring as a marker for educational performance simulation. | P < 0.0001 | Spinal Neurosurgery | 13 | [35] |
| Kirkman et al 2015 | 2015 | UK | Cohort Prospective Study | Medtronic StealthStation S7 Surgical Navigation System, Axiem Cranial Non-invasive kit | Medtronic StealthStation S7 Surgical Navigation System, AxiEM Cranial Non-Invasive Shunt Kit, reporting a inversely correlated performance with both objective and self-reported measures of stress, Pro-Form Strapless Heart Rate Monitor Watch | p < 0.001 | Cranial Neurosurgery: Hydrocephalus, ICP | 12 | [57] |
| Zammer et al 2015 | 2015 | USA | Cohort Study: Survey Based Design | Cerebral angiography simulator | Anastomosis and diagnostic cerebral angiography, assessment of three facets including pre-didactic cognitive knowledge and technical skills testing, didactic lecture based, Post didactic cognitive knowledge and technical skills testing | P < 0.01 | Cranial Neurosurgery: Neurovascular | 11 | [125] |
| Li et al 2015 | 2015 | CHINA | RCT | 3D printer (XYZ printing) | 3D Printing/ Spinal Neurosurgery/ Medical Students improvement in efficiency |  | Spinal Neurosurgery | 13.5 | [60] |
| Shakur et al 2015 | 2015 | USA | Cohort Study: Experimental Design | ImmersiveTouch haptics-based virtual reality percutaneous trigeminal rhizotomy simulator | Assessment of usefulness of a novel haptics-based virtual reality percutaneous trigeminal rhizotomy simulator, construct validity of the simulator, increasing PGY level is significantly associated with a decreased distance from the ideal entry point and a better final score. | p=0.007 | Cranial Neurosurgery: Functional | 13 | [106] |
| Holloway et al 2015 | 2015 | USA | Cohort Study: Experimental Design | Virtual reality brain surgery NeuroTouch simulator environment | Study used to develop measures to differentiate between experienced and inexperienced neurosurgeons in a virtual reality brain surgery NeuroTouch simulator environment, Medical Students vs Residents, Glioblastoma Multiforme resections, volume of tumor removed, volume of healthy brain removed, and instrument path length (mm) were recorded. Additionally, surgical effectiveness (% tumor removed divided by % healthy brain removed) and efficiency. Significant Improvement in removal of Tumour volume by medical students. | p<0.001 | Cranial Neurosurgery: Neurooncology | 12 | [48] |
| Alaraj et al 2015 | 2015 | USA | Cohort Study: Experimental Design | Immersive Touch platform haptic-based virtual reality simulator | Study used to develop and evaluate the usefulness of a Immersive Touch platform for open surgery haptic-based virtual reality simulator in the training of neurosurgical residents, middle cerebral artery aneurysms | - | Cranial Neurosurgery: Neurovascular | 7.5 | [1] |
| Shah et al 2016 | 2016 | USA | Cohort Study: Assessment-based | uPrint Stratasys 3D printer and Stereolithography with thermoplastic material | stereolithography, or 3-dimensional (3-D) printing as a valid educational tool Scoring was based on correctly naming the anatomical structure, Improvement for Groups B 7.5 after the lecture and 3-D model simulation | P = 0.002 | Cranial Neurosurgery: Skullbase | 10.5 | [104] |
| Clarke et al 2016 | 2016 | CANADA | RCT | PeriopSim Instrument Trainer | PeriopSim Instrument Trainer and PeriopSim for Burhole Training, game-based simulation | p < 0.0005 | Cranial Neurosurgery | 14.5 | [19] |
| Perin et al 2016 | 2016 | ITALY | Experimental Pilot Study | USIM for Intraoperative Ultrasound-related neurosurgical simulation | USIM for Intraoperative Ultrasound-related neurosurgical simulation for rehearsal before operation. | p<0.008 | Cranial Neurosurgery | 10 | [83] |
| Rooney et al 2016 | 2016 | USA | Cohort Study: Assessment-based | Ventriculostomy Simulator | Validation and standardising of performance measures, Ventricolostomy Procedural Assessment Tool items correlation with Objective Structured Assessment for Technical Skills, targeting ventriculostomy simulators, those currently on the market are virtual reality (VR)- | p<0.01 | Cranial Neurosurgery: Hydrocephalus, ICP | 12.5 | [97] |
| Ryan et al 2016 | 2016 | USA | Cohort Study Survey Based Design | Likert Scale Evaluation, zPrinter 3D Printing | patient-derived medical simulacrum, 3DSystems 3D printing with zPrinter 650 of vascular and brain models, 7 question survey on Likert scale testing Efficacy |  | Cranial Neurosurgery: Neurovascular | 7 | [98] |
| Sundar et al 2016 | 2016 | USA | RCT | Cephalus to pelvis cadaveric specimens | Lab based spinal fixation using navigation software combined with cadaveric specimens and accessibility to Sawbones models. | p<0.02 | Spinal Neurosurgery | 13.5 | [111] |
| Marcus et al 2016 | 2016 | UK | Cohort Study: Laboratory In-Vivo Experimental Study | “smart” force-limiting instrument for microsurgery, OSAT | A Laboratory based experimental design study to validate smart force limiting instrument for microsurgery with vibrotactile feedback, microsurgical dissection, Primary Outcomes were forces exerted and OSAT Scores | p<0.007 | Cranial Neurosurgery | 13.5 | [67] |
| Thawani et al 2016 | 2016 | USA | Cohort Study: Experimental Design | NeuroTouch haptic simulation platform | A study of improvement in resident performance in endoscopic endonasal surgery. Using the NeuroTouch haptic simulation platform, Post-simulation evaluation over 6months to gauge improvement | p=0.0045 | Cranial Neurosurgery: Pituitary | 12.5 | [114] |
| Nakhla et al 2017 | 2017 | USA | Experimental Pilot Study | Google Glass technology | Google Glass technology for Neurosurgical simulation, Wearable technology to capture video segments for trainees to review |  | General Neurosurgery | 7 | [77] |
| Chugh et al 2017 | 2017 | USA | RCT | Surgical rehearsal platform SuRgical Planner (SRP) | Improvement and efficiency in Aneurysm Clipping Time using SRP, time per clip use. Surgical rehearsal platform SuRgical Planner (SRP) | p<0.05 | Cranial Neurosurgery: Neurovascular | 15 | [17] |
| Boody et al 2017 | 2017 | USA | RCT | Questionnaire assessment | Effectiveness of OSAT assessment tool, Mean Improvement in OSATS and PPDIS | p<0.0001 | Spinal Surgery | 13.5 | [7] |
| Breimer et al 2017a | 2017 | USA | Cohort Study: Experimental Design | VR ETV simulation model | Compare and identify the relative utility of a physical and VR ETV simulation model for use in neurosurgical training, 5 point likert scale evaluation of the domains of anatomy, instrument handling, procedural content, and the overall fidelity of the simulation. Physical simulator outperformed VR simulation | p < 0.001 | Cranial Neurosurgery: Hydrocephalus, ICP | 13 | [8] |
| Breimer et al 2017b | 2017 | USA | Cohort Study: Assessment-based | S.I.M.O.N.T Neurosurgical Endotrainer and  NEVAT assessment tool | Provide validity evidence of the NEVAT by reporting on the tool’s internal structure and its relationship with surgical expertise during simulation-based training in experts, experienced trainees and novices. | P = .04 | Cranial Neurosurgery Hydrocephalus, ICP |  | [9] |
| Xin et al 2018 | 2018 | CHINA | RCT | Immersive Virtual Reality Surgical Simulator | Improvement of performance/safety/Accuracy/Efficacy, Immersive Virtual Reality Surgical Simulator Training IVRSS-PSP) Spinal Neurosurgery | p<0.05 | Spinal Neurosurgery |  | [122] |
| Perin et al 2018 | 2018 | ITALY | Cohort Study: Experimental Study | ImmersiveTouch simulation platform | Accuracy of EVD placement using a high-resolution and high-performance virtual reality platform with haptic feedback technology, measured placement of catheter was inside the ventricle, we recorded the distance from the tip of the tool to the homolateral foramen of Monro. |  | Cranial Neurosurgery: Hydrocephalus, ICP |  | [82] |
| Santangelo et al 2018 | 2018 | USA | Cohort Study Experimental Design | High Fidelity Whole task simulator Carotid endarterectomy, 3-dimensional (3D) printing and polyvinyl alcohol (PVA) hydrogels | High Fidelity Whole task simulator Carotid endarterectomy, 3-dimensional (3D) printing and polyvinyl alcohol (PVA) hydrogels, measured outcome of mean operative time for the expert group was 63.6 min vs 138.8 for the resident group (P = .002). There was a difference in mean internal carotid artery clamp time of 43.4 vs 83.2 min (P = .04). There were only 2 hypoglossal nerve injuries, both in the resident group (P = .009). | p<0.002 | Cranial Neurosurgery: Neurovascular | 11.5 | [99] |
| Bughadi et al 2018 | 2018 | CANADA | Cohort Study: Model Based | NeuroVR (formerly NeuroTouch) | Testing the Fitts and Posner Model of motor learning, simulated brain tumour resection in Nine neurosurgeons, 10 senior residents, and 8 junior residents. |  | Cranial Neurosurgery: Neurooncology |  | [12] |
| Grillo et al 2018 | 2018 | USA | Cohort Study: Image-based study | 3D printing by the Paragon device (Rapid Technologies) | 3D model and 3D printing by the Paragon device of a child with Sturge Webber syndrome, image-guided neuronavigation (IGN) with tissue-mimicking materials to create a patient specific phantom for procedural simulation, InVesalius software with Blender to create the 3D model | p < 0.001 | Cranial Neurosurgery: Neurovascular | 7 | [39] |
| Hanrahan et al 2018 | 2018 | UK | Cohort Study: Survey Based Design | Ex vivo pig model | Simulation based learning workshop instructing students to insert an intracranial pressure bolt using an ex vivo pig model | p < 0.001 | Cranial Neurosurgery: Hydrocephalus, ICP | 11.5 | [43] |
| Wang et al 2018 | 2018 | CHINA | Cohort Study: Prospective Study/Survey based | 3D printing simulation model using Connex Multi-Material 3D Printer (MoonRay) | Mimics reconstruction 3D-DSA in patients, simulation models including intracranial aneurysmal and parent vessel geometries, as well as vascular branches, through 3D printing technology in patients by injecting iopamidole, residents |  | Cranial Neurosurgery: Neurovascular | 7 | [117] |
| Sun and Qi 2018 | 2018 | CHINA | RCT | Adult Lumbar Puncture simulator (ZH-L260B), Pre-test and Post-test questionairres | Improvement procedural competence, Pretest and Post-test, Lumbar Puncture Simulator/Neurosurgery/Neurology | p<0.001 | Spinal Neurosurgery | 11 | [110] |
| Hou et al 2018 | 2018 | CHINA | RCT | Cadeveric Spinal Specimen | Virtual Reality and Cadaveric/Spine Neurosurgery, improvement in efficiency | p<0.05 | Spinal Neurosurgery | 13.5 | [51] |
| Sarwaya et al 2018 | 2018 | CANADA | Cohort Study: Experimental Design | NeuroVR Virtual reality | Quadrantized Assessment of Hand ergonomics, following resection of simulated brain tumors on the NeuroVR, spatial distribution of time expended, force applied, and tumor volume removed was analyzed | p < 0.01 | Cranial Neurosurgery: Neurooncology | 10 | [100] |
| Buchanan et al 2019 | 2019 | USA | Cohort Study Cadaveric Study | Perfusion-based cadaveric Model, MISS Durotomy Repair | Achieving Dural Repair in Perfusion-based cadaveric Model, MISS Durotomy Repair in neurosurgery residents | p<0.02 | Cranial Neurosurgery | 11 | [11] |
| Bairamian et al.2019 | 2019 | USA | Cohort Study: Experimental Design | Dreamer Flashforge 3D Model, an Android smartphone, and the Google Daydream R headset (Alphabet Inc) | Investigated and compared the practicality and potential of 3D printed and VR models in a neurosurgical education context, trainees rated 9 aspects of their experience with 3D printed and VR models in a Likert-like questionnaire. Depth of Perception and ease of manipulation | P = 0.007 | Cranial Neurosurgery: Neurovascular | 12.5 | [4] |

***Supplementary Table 1***

| **Author** | **Year** | **Location** | **Design** | **Type of Simulation** | **Population** | **Analysis Domain** | **Outcome** | **Ne** | **Nc** | **Me** | **Se** | **Mc** | **Sc** | **Ref** |
| --- | --- | --- | --- | --- | --- | --- | --- | --- | --- | --- | --- | --- | --- | --- |
| Boody et al 2017 | 2017 | USA | Prospective Randomised Design | OSAT score/Spinal Sawbones Model | Students to PostgradYear5 Residents | Improvement in knowledge and procedural skill | OSAT score | 11 | 9 | 6.23 | 4.474 | 0.889 | 3.855 | [7] |
|  | | | | | | | PPDIS score | 11 | 9 | 5.545 | 2.66 | 0.112 | 0.354 |  |
|  |  |  |  |  |  |  | ODS score | 11 | 9 | 6.364 | 4.945 | 2.22 | 2.33 |  |
| Chugh et al 2017 | 2017 | USA | Prospective RCT | Surgical rehearsal platform/Vascular Neurosurgery | Patients/Surgeons | Improvement of safety | Aneurysm Clipping Time using SRP | - | - | - | - | - | - | [17] |
|  | | | | | | Improvement of safety | Clip Attempts | 14 | 11 | 5.07 | 2.78 | 6.09 | 3.7 |  |
|  |  |  |  |  |  | Improvement of safety | Clip Usage | 14 | 11 | 1.79 | 0.97 | 1.36 | 0.67 |  |
|  |  |  |  |  |  | Improvement in speed | Total Time | 14 | 11 | 1320 | 884 | 1521 | 598 |  |
|  |  |  |  |  |  | Improvement of safety | Ratio of Clip attempt to Clip Usage | 14 | 11 | 3.13 | 2.19 | 4.97 | 3.7 |  |
|  |  |  |  |  |  | Improvement in speed | Time per clip used | 14 | 11 | 920 | 770 | 1294 | 678 |  |
|  |  |  |  |  |  | Improvement in speed | Time per clip attempt | 14 | 11 | 321 | 223 | 376 | 300 |  |
| Hou et al | 2018 | China | Randomised Design | Virtual Reality and Caderveric/Spine Neurosurgery | Residents | Improvement of accuracy | Screw Penetration Distance and accuracy | 70 | 70 | 1.23 | 0.56 | 2.37 | 0.23 | [51] |
| Li et al 2015 | 2015 | China | Prospective RCT | 3D Printing/ Spinal Neurosurgery | Medical Students | Improvement of knowledge | **Measure of Understanding 3d images vs Control Male** | 19 | 22 | 506.89 | 175.61 | 708.56 | 212.08 | [60] |
|  | | | | | | Improvement of knowledge and Understanding | Measure of Understanding 3d images vs Control female | 22 | 22 | 593.48 | 207.36 | 896.95 | 266.08 |  |
|  |  |  |  |  |  | Improvement of knowledge and Understanding | Measure of Understanding 3d printed vs Control male | 18 | 18 | 373.56 | 206.97 | 708.56 | 212.08 |  |
|  |  |  |  |  |  | Improvement of knowledge and Understanding | Measure Understanding 3d printed vs Control Female | 22 | 22 | 376.73 | 138.95 | 896.95 | 266.08 |  |
|  |  |  |  |  |  | Improvement of knowledge | **Measure of Knowledge improvement 3d images vs Control Male** | 19 | 18 | 7.16 | 1.71 | 4.28 | 1.45 |  |
|  |  |  |  |  |  | improvement of knowledge | Measure of Knowledge improvement 3d images vs Control Female | 22 | 21 | 5.76 | 1.26 | 3.95 | 1.17 |  |
|  |  |  |  |  |  | improvement of knowledge | Measure of Knowledge improvement 3d printed vs Control Male | 18 | 18 | 7.17 | 1.34 | 4.28 | 1.45 |  |
|  |  |  |  |  |  | improvement of knowledge | Measure of Knowledge improvement 3d printed vs Control Female | 22 | 22 | 7.18 | 1.53 | 3.95 | 1.17 |  |
| Sun and Qi 2018 | 2018 | China | Randomised Design | Lumbar Puncture Simulator/Neurosurgery/Neurology | Residents | Improvement knowledge and procedural skill | Measure of Success in Performing Skill in PSBL vs Traditional Methods(control) |  |  |  |  |  |  | [110] |
|  | | | | | | Improvement knowledge and procedural skill | Pretraining | 30 | 30 | 1.33 | 0.61 | 1.3 | 0.53 |  |
|  |  |  |  |  |  | Improvement knowledge and procedural skill | Posttraining | 30 | 30 | 3.7 | 0.92 | 2.67 | 0.84 |  |
|  |  |  |  |  |  | Improvement knowledge and procedural skill | Succesful LP performed 1year after training (n=10)? | 30 | 30 | 8.4 | 0.86 | 7.7 | 0.99 |  |
|  |  |  |  |  |  | Improvement knowledge and procedural skill | Improvement Rate | 30 | 30 | 12.73 | 3.76 | 11.13 | 3.61 |  |
|  |  |  |  |  |  | Improvement knowledge and procedural skill | Pretest | 30 | 30 | 6.53 | 3.03 | 7.27 | 3.18 |  |
|  |  |  |  |  |  | Improvement knowledge and procedural skill | Posttest | 30 | 30 | 19.27 | 1.93 | 18.4 | 1.92 |  |
| Sundar et al 2016 | 2016 | USA | RCT Single Blinded | Virtual Reality Software combined with Cadervaric, Spinal Neurosurgery | Residents/ Students | Improvement of safety | Reduction in surgical error | 5 | 5 | - | - | - | - | [111] |
|  | | | | | | Improvement of safety | cervical level surgical error | 1 | 5 | - | - | - | - |  |
|  |  |  |  |  |  | Improvement of safety | thoracic level surgical error | 9 | 34 | - | - | - | - |  |
|  |  |  |  |  |  | Improvement of safety | Lumbar level surgical error | 8 | 17 | - | - | - | - |  |
|  |  |  |  |  |  | Improvement of safety | Sacral level surgical error | 3 | 3 | - | - | - | - |  |
|  |  |  |  |  |  | Improvement of safety | Total Number of Errors Overall | 21 | 59 | - | - | - | - |  |
|  |  |  |  |  |  | Improvement of safety | Total Number of Errors Overall with suboptimally placed screws | 37 | 78 | - | - | - | - |  |
|  |  |  |  |  |  | Improvement of safety | Major Errors | 10 | 25 | - | - | - | - |  |
|  |  |  |  |  |  | Improvement of safety | Minor Errors | 11 | 34 | - | - | - | - |  |
|  |  |  |  |  |  | Improvement of safety | Comfort level of Screw placement before | 5 | 5 | - | - | - | - |  |
|  |  |  |  |  |  | Improvement of safety | comfort level in Cervical 1 screw placement | 5 | 5 | 1.2 | 0.5 | 1.2 | 0.5 |  |
|  |  |  |  |  |  | Improvement of safety | comfort level in Cervical 2 screw placement | 5 | 5 | 1.5 | 0.6 | 1.5 | 0.6 |  |
|  |  |  |  |  |  | Improvement of safety | comfort level in Cervical 3-6 screw placement | 5 | 5 | 2 | 1.4 | 1.8 | 0.8 |  |
|  |  |  |  |  |  | Improvement of safety | comfort level in Cervical 7 screw placement | 5 | 5 | 1.6 | 0.9 | 1.4 | 0.6 |  |
|  |  |  |  |  |  | Improvement of safety | Thoracic comfort in screw placement | 5 | 5 | 1.8 | 1.1 | 1.8 | 1.1 |  |
|  |  |  |  |  |  | Improvement of safety | Lumbar comfort in screw placement | 5 | 5 | 2.2 | 1.1 | 2.4 | 1.5 |  |
|  |  |  |  |  |  | Improvement of safety | comfort level in Sacral 1 screw placement | 5 | 5 | 1.4 | 0.9 | 2 | 1.4 |  |
|  |  |  |  |  |  | Improvement of safety | comfort level in Sacral 2 screw placement | 5 | 5 | 1 | 0 | 1.6 | 0.9 |  |
|  |  |  |  |  |  | Improvement of safety | iliac level comfort in screw placement | 5 | 5 | 1.2 | 0.5 | 1.6 | 1.3 |  |
| Xin et al 2018 | 2018 | China | RCT, Double blind | Immersive Virtual Reality Surgical Simulator Training IVRSS-PSP) Spinal Neurosurgery | Residents/ Students | Improvement of safety | **Accuracy of pedicle screw placement** | - | - | - | - | - | - | [122] |
|  | | | | | | Improvement of speed | Total Time of pedical screw placement | 48 | 48 | 2.8 | 1 | 4.9 | 1 |  |
| Patel et al 2014 | 2014 | USA | Randomised Design | Tactile Simulation using Virtual Reality, Simulation vs no simulation/ using object size | Students | Improvement of safety | Localisation before sim training vs no sim training | 10 | 10 | - | - | - | - | [81] |
|  | | | | | | Improvement of safety | object localisation sim vs no sim | 10 | 10 | - | - | - | - |  |
| Clarke et al 2016 | 2016 | Canada | RCT | PeriopSimTM instrument trainer | Resident Surgeons | Improvement in speed | Improvement in Time saved/Efficiency | - | - | - | - | - | - | [19] |

***Supplementary Table 2***
