# Supplementary material for: Simulation for skills training in neurosurgery: a systematic review, meta-analysis, and analysis of progressive scholarly acceptance
Source: Neurosurg Rev. 2020 Sep 18;44(4):1853–67. doi: 10.1007/s10143-020-01378-0 (PMC8338820; doi:10.1007/s10143-020-01378-0)
Supplement: Supplementary file 2 — (PDF 294 kb) [file 10143_2020_1378_MOESM2_ESM.pdf]

MAIN MANUSCRIPT FIGURES

LIST OF FIGURE CAPTIONS

Fig 1 PRISMA flow chart *of the systematic process utilised for the review. Fifty-six articles were included in the final qualitative review with 32 in a quantitative meta-analysis including 7 randomised studies measuring various outcome domains.*

Fig 2 Knowledge Meta-analysis a) *Forest plot (top), outcome measures with focus primarily on knowledge assessment as a simulation domain.* ***b)*** *Forest plot (middle) outcome measure of procedural knowledge linked with using scoring systems for simulation.* ***c)*** *Forest Plot (bottom) of pooled studies investigating the outcome measure of knowledge together with procedural skill performed on various simulator types.*

Fig 3 Accuracy Meta-analysis *Forest Plot of pooled studies investigating outcome measure of accuracy. Supplementary data summarises the measures calculated for this domain in simulation and metanalysis results of measured improvement in accuracy. A random effect model as shown. Statistical significance set at $P < 0.05$.*

Fig 4 Speed meta-analysis a) *Additional data summarising the statistical analysis of the model estimates; a 3.95 times speed improvement from simulation was seen (top).* ***b****) Forest Plot showing pooled studies (bottom) analysing the outcome measure of speed as time to task completion. Some studies assessed speed using a surgical rehearsal platform and instrument trainer platform simulators, a marginal improvement in speed is seen.*

Supplementary Fig 1 Miscellaneous Meta-analysis on other metrics*: Significant moderately heterogeneous metanalysis of studies demonstrating improvement of other objectively measured safety parameters as outcome domains.*

Supplementary Fig 2 *Metanalysis of other safety domains studied included a) fluoroscopy shots (top), b) comfort (bottom).*

Supplementary Fig 3 *Cochrane Risk of Bias assessment for randomised trials****.*** *Inadequate standardisation protocols meant methods for blinding and reporting bias appeared prevalent in most of these studies.*

Supplementary Fig 4 PSA literature results*. PSA as a metric to gauge adoption of VR simulation in neurosurgical educational practice over a 20-year timeframe (1998-2018).* ***a)*** *Annual publication of initial (thin black) and refining (thick grey) reports.* ***b)*** *The number of annual initial (thin black) and*

refining (thick grey) studies compounded each year. (Adapted method from Schnurman & Kondziolka 2016).

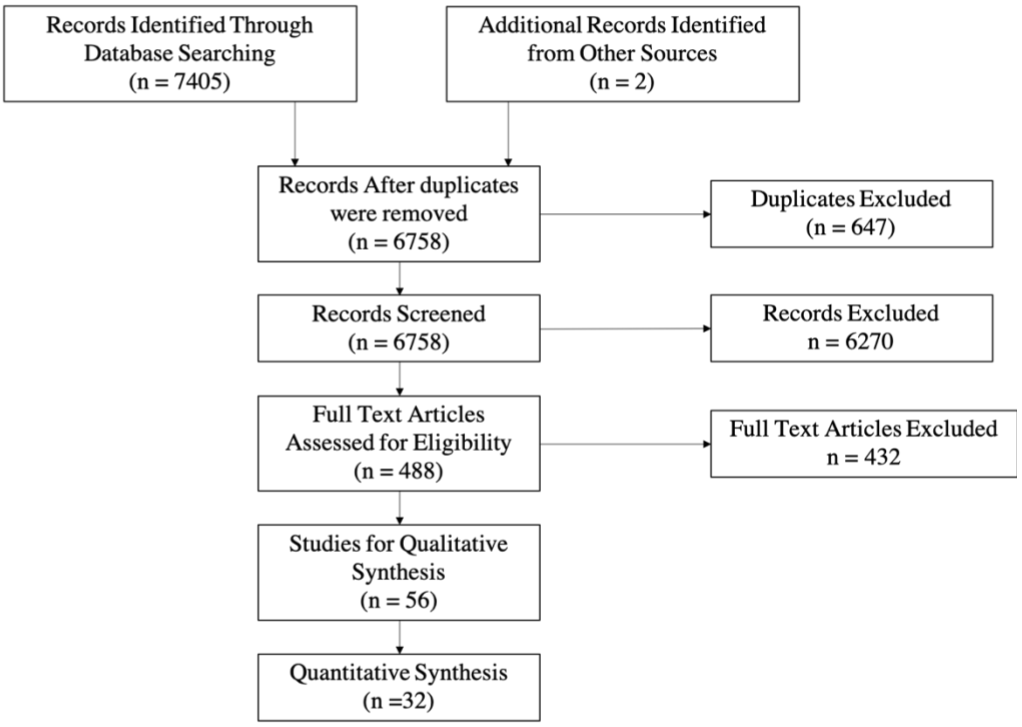

Fig 1 PRISMA flow chart

Knowledge only

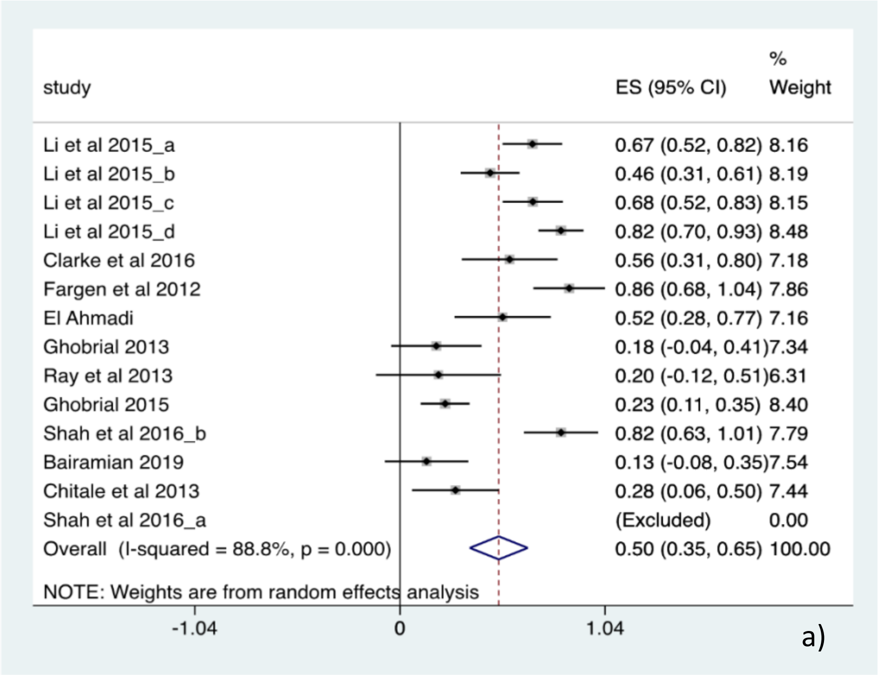

Knowledge using scores

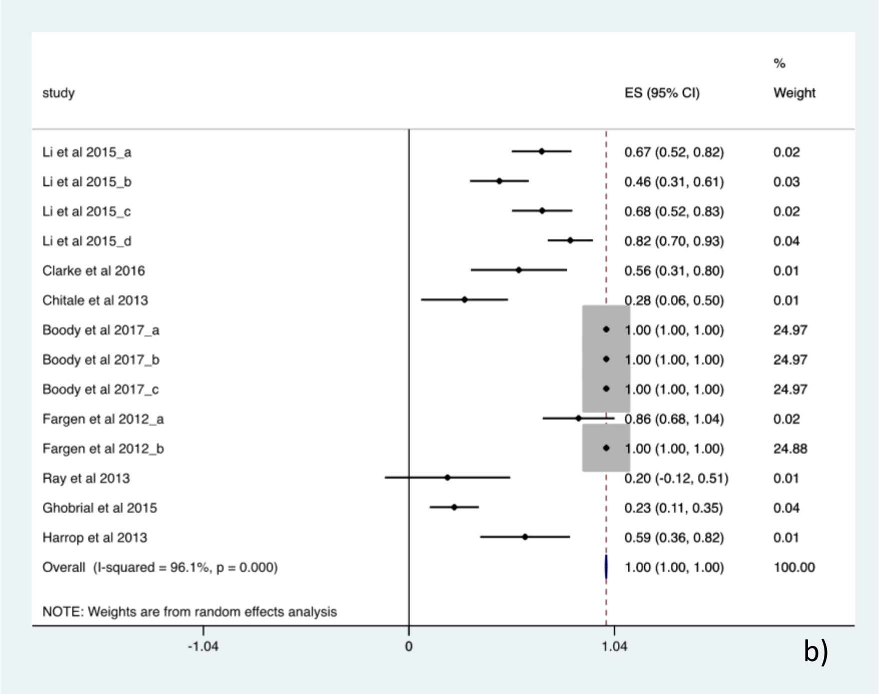

Knowledge pooled

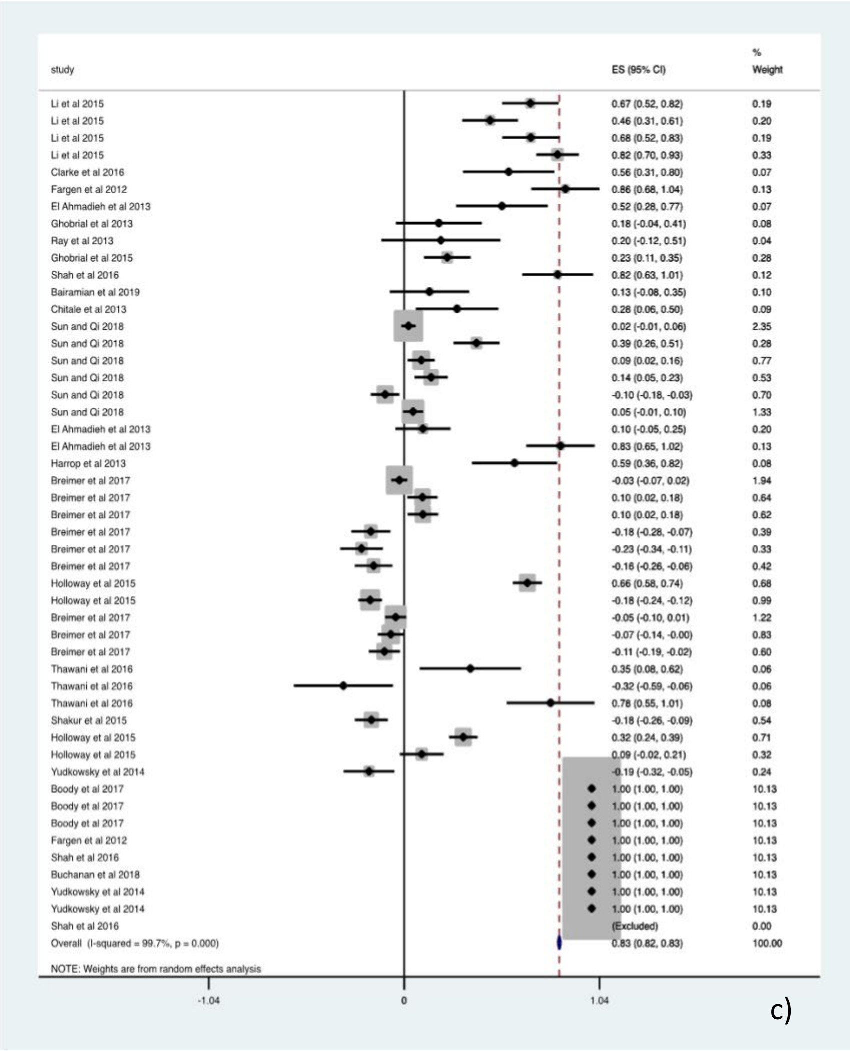

Fig 2 Knowledge Meta-analysis

Accuracy

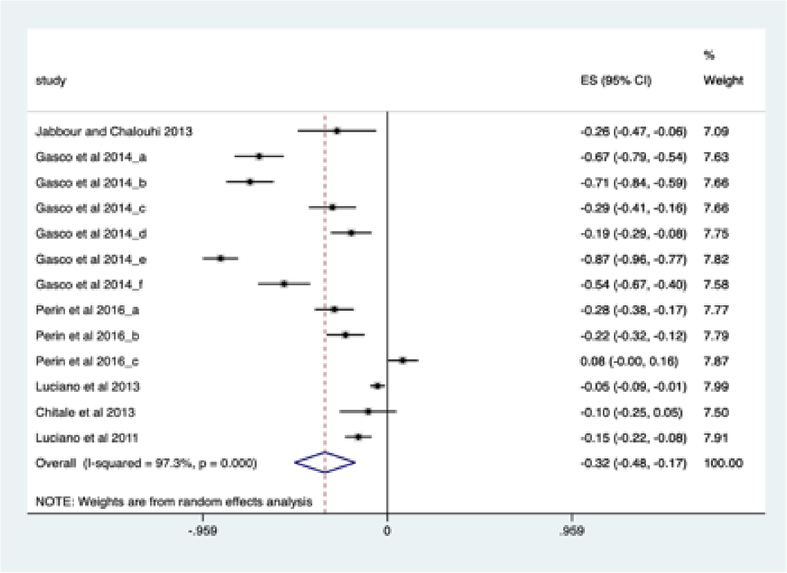

Fig 3 Accuracy Meta-analysis

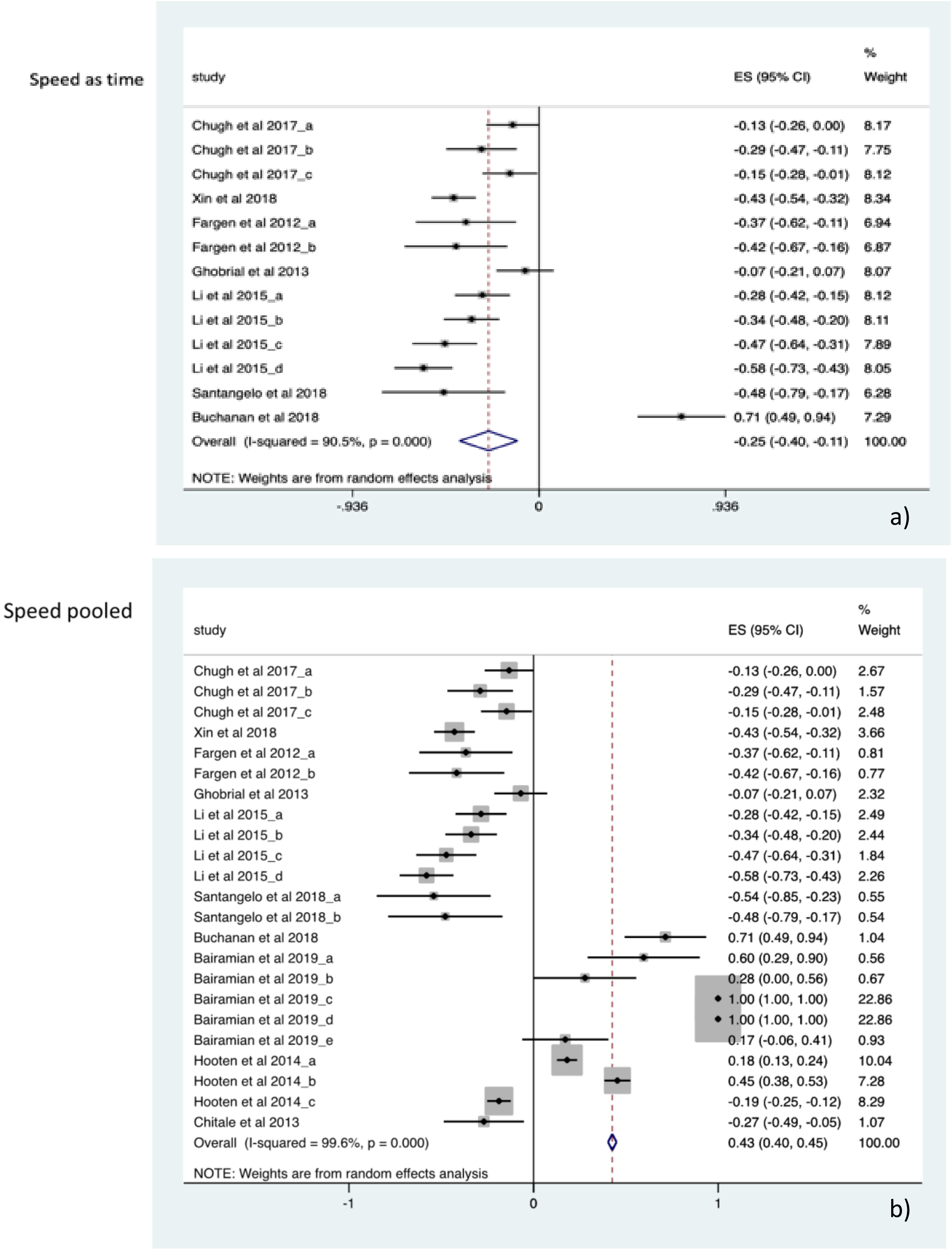

Fig 4 Speed meta-analysis

SUPPLEMENTARY FIGURES

Safety others

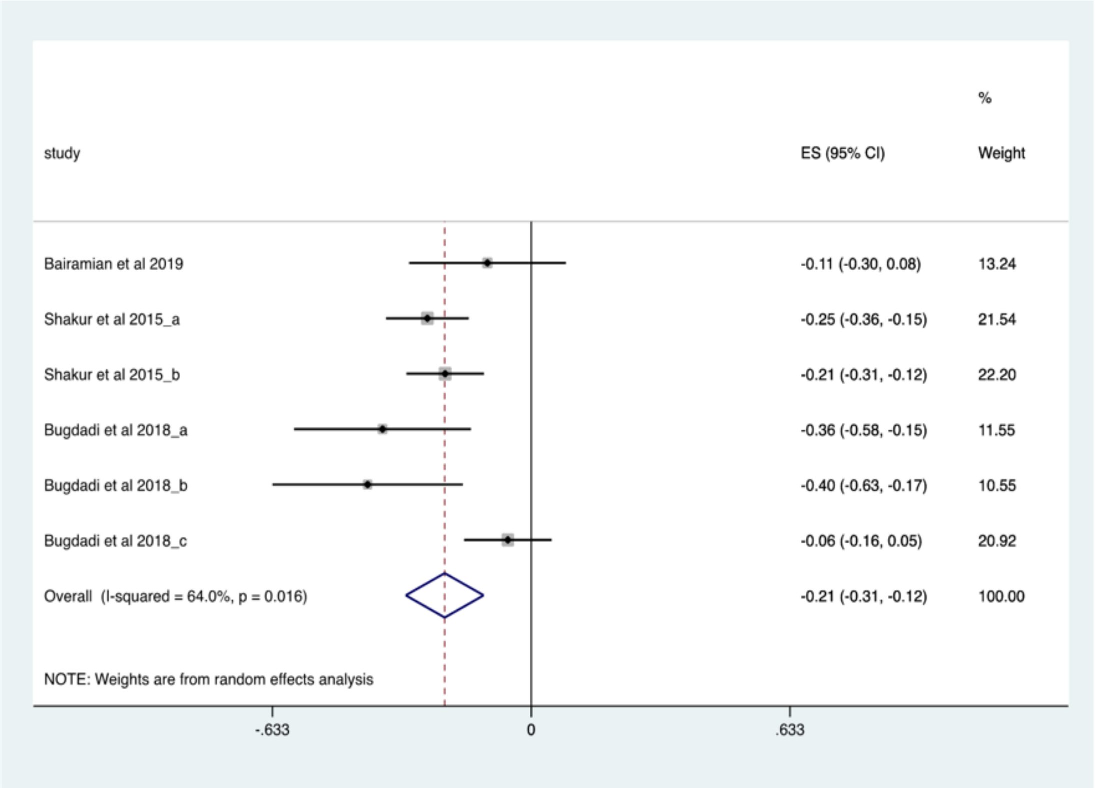

Supplementary Fig 1

Safety fluoroscopy shots

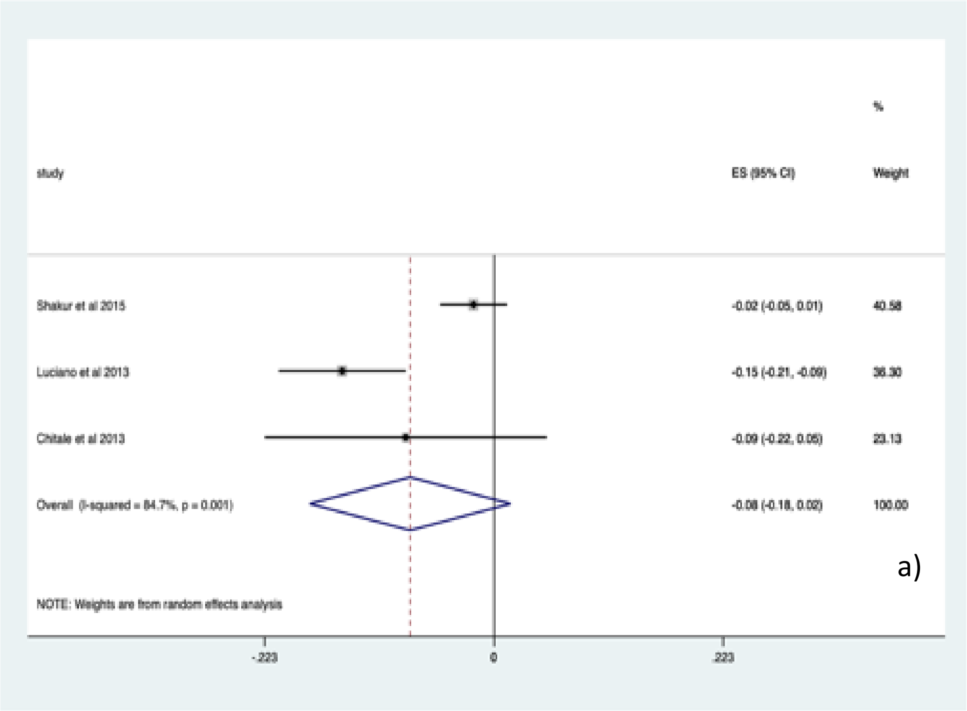

Safety comfort

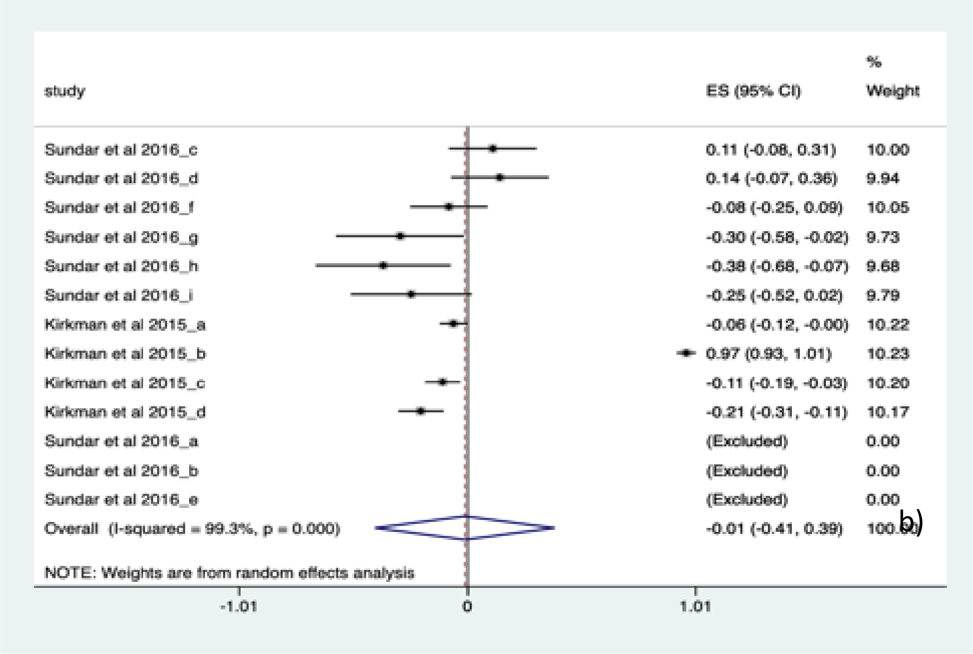

Supplementary Fig 2

Author	Random Sequence Generation	Allocation Concealment	Blinding of Participants & Personnel	Blinding of Outcome Assessment	Incomplete Outcome data	Selective Reporting	Other Bias
Bekeles et al 2017	+	?	+	?	?	?	?
Chugh et al 2017	+	?	+	?	+	-	?
Boody et al 2017	+	?	+	+	+	+	+
Clarke et al 2016	+	+	+	?	?	+	?
Sun & Chi 2018	+	?	+	?	+	?	-
Sundar et al 2016	+	-	+	?	?	?	?
Xin et al 2018	+	+	+	?	+	+	?
Patel et al 2014	?	-	-	?	+	?	-

Bias assessments for randomised studies using Cochrane risk of bias tool.

Supplementary Fig 3

a) b)

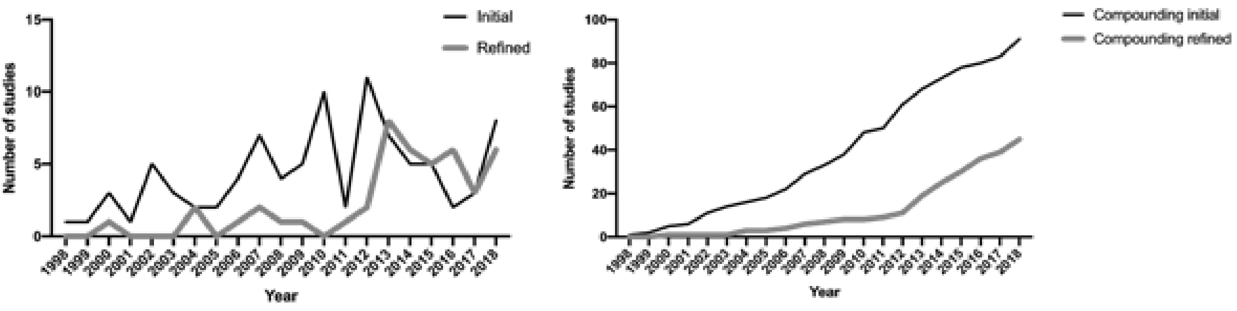

Supplementary Fig 4:
